# Human induced pluripotent stem cell–derived atrial cardiomyocytes recapitulate contribution of the slowly activating delayed rectifier currents *I*_Ks_ to repolarization in the human atrium

**DOI:** 10.1093/europace/euae140

**Published:** 2024-05-24

**Authors:** Muhammed Ikbal Sönmez, Silvana Goldack, Elina Nurkkala, Carl Schulz, Birgit Klampe, Thomas Schulze, Arne Hansen, Thomas Eschenhagen, Jussi Koivumäki, Torsten Christ

**Affiliations:** Institute of Experimental Pharmacology and Toxicology, University Medical Center Hamburg-Eppendorf, Martinistraße 52, 20246 Hamburg, Germany; DZHK (German Centre for Cardiovascular Research), partner site Hamburg/Kiel/Lübeck, Martinistrasse 52, 20246 Hamburg, Germany; Institute of Experimental Pharmacology and Toxicology, University Medical Center Hamburg-Eppendorf, Martinistraße 52, 20246 Hamburg, Germany; DZHK (German Centre for Cardiovascular Research), partner site Hamburg/Kiel/Lübeck, Martinistrasse 52, 20246 Hamburg, Germany; Department of Pharmacology and Toxicology, Medical Faculty Carl Gustav Carus, Dresden University of Technology, Dresden, Germany; Tech Unit and Centre of Excellence in Body-on-Chip Research (CoEBoC), Faculty of Medicine and Health Technology, Tampere University, Tampere, Finnland; Institute of Experimental Pharmacology and Toxicology, University Medical Center Hamburg-Eppendorf, Martinistraße 52, 20246 Hamburg, Germany; DZHK (German Centre for Cardiovascular Research), partner site Hamburg/Kiel/Lübeck, Martinistrasse 52, 20246 Hamburg, Germany; Institute of Experimental Pharmacology and Toxicology, University Medical Center Hamburg-Eppendorf, Martinistraße 52, 20246 Hamburg, Germany; DZHK (German Centre for Cardiovascular Research), partner site Hamburg/Kiel/Lübeck, Martinistrasse 52, 20246 Hamburg, Germany; Institute of Experimental Pharmacology and Toxicology, University Medical Center Hamburg-Eppendorf, Martinistraße 52, 20246 Hamburg, Germany; DZHK (German Centre for Cardiovascular Research), partner site Hamburg/Kiel/Lübeck, Martinistrasse 52, 20246 Hamburg, Germany; Institute of Experimental Pharmacology and Toxicology, University Medical Center Hamburg-Eppendorf, Martinistraße 52, 20246 Hamburg, Germany; DZHK (German Centre for Cardiovascular Research), partner site Hamburg/Kiel/Lübeck, Martinistrasse 52, 20246 Hamburg, Germany; Institute of Experimental Pharmacology and Toxicology, University Medical Center Hamburg-Eppendorf, Martinistraße 52, 20246 Hamburg, Germany; DZHK (German Centre for Cardiovascular Research), partner site Hamburg/Kiel/Lübeck, Martinistrasse 52, 20246 Hamburg, Germany; Tech Unit and Centre of Excellence in Body-on-Chip Research (CoEBoC), Faculty of Medicine and Health Technology, Tampere University, Tampere, Finnland; Institute of Experimental Pharmacology and Toxicology, University Medical Center Hamburg-Eppendorf, Martinistraße 52, 20246 Hamburg, Germany; DZHK (German Centre for Cardiovascular Research), partner site Hamburg/Kiel/Lübeck, Martinistrasse 52, 20246 Hamburg, Germany

**Keywords:** Sinus rhythm, Atrial fibrillation, hiPSC-aCM, Atrial EHT, *I*_Ks_, Action potential, Repolarization reserve, Computer model

## Abstract

**Aims:**

Human induced pluripotent stem cell–derived atrial cardiomyocytes (hiPSC-aCM) could be a helpful tool to study the physiology and diseases of the human atrium. To fulfil this expectation, the electrophysiology of hiPSC-aCM should closely resemble the situation in the human atrium. Data on the contribution of the slowly activating delayed rectifier currents (*I*_Ks_) to repolarization are lacking for both human atrium and hiPSC-aCM.

**Methods and results:**

Human atrial tissues were obtained from patients with sinus rhythm (SR) or atrial fibrillation (AF). Currents were measured in human atrial cardiomyocytes (aCM) and compared with hiPSC-aCM and used to model *I*_Ks_ contribution to action potential (AP) shape. Action potential was recorded by sharp microelectrodes. HMR-1556 (1 µM) was used to identify *I*_Ks_ and to estimate *I*_Ks_ contribution to repolarization. Less than 50% of hiPSC-aCM and aCM possessed *I*_Ks_. Frequency of occurrence, current densities, activation/deactivation kinetics, and voltage dependency of *I*_Ks_ did not differ significantly between hiPSC-aCM and aCM, neither in SR nor AF. β-Adrenoceptor stimulation with isoprenaline did not increase *I*_Ks_ neither in aCM nor in hiPSC-aCM. In tissue from SR, block of *I*_Ks_ with HMR-1556 did not lengthen the action potential duration, even when repolarization reserve was reduced by block of the ultra-rapid repolarizing current with 4-aminopyridine or the rapidly activating delayed rectifier potassium outward current with E-4031.

**Conclusion:**

*I*_Ks_ exists in hiPSC-aCM with biophysics not different from aCM. As in adult human atrium (SR and AF), *I*_Ks_ does not appear to relevantly contribute to repolarization in hiPSC-aCM.

## Graphical Abstract

**Graphical Abstract:**
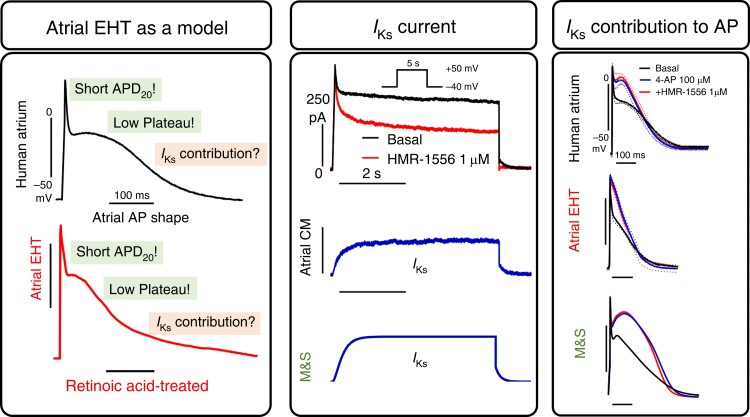

**What’s new?**

- Atrial human induced pluripotent stem cell–derived atrial cardiomyocytes (hiPSC-CM) possess IKs.
- Biophysics of IKs in atrial hiPSC-CM closely resembles the situation in the human atrium.
- Action potential shape in the human atrium and in hiPSC-aCM does not favour relevant contribution of IKs.

## Introduction

Atrial fibrillation (AF) remains a challenge.^1,2^ The electrophysiological characteristics of chronically remodelled atria (several months) have been studied in several *in vitro* studies using human tissues collected during open-heart surgery. Early adaptation to rapid activation of the atria (within hours or a few days) cannot be addressed by measuring action potential (AP) from patients that were mostly in AF for more than 6 months. Furthermore, AF is strongly associated with single nucleotide polymorphism (SNP), but the functional relevance of the SNPs is poorly understood. Human-induced pluripotent stem cell–derived atrial cardiomyocytes (hiPSC-aCM)^3–5^ are expected to add substantially to both fields.^6,7^ However, the value of hiPSC-aCM for studying human atrial electrophysiology will critically depend on how closely hiPSC-aCM resemble the electrophysiological properties of human SR atrium cardiomyocytes.

Supplementation of cardiac differentiation protocols with retinoic acid (RA) has been shown to induce an atrial phenotype in hiPSC-CM.^3,4^ Recently, we showed that the concentration of RA is critical to induce a fully human atrium–like AP shape in hiPSC-aCM, characterized by strong contribution of two atrial-selective potassium currents: the ultra-rapid activating delayed rectifier potassium current (*I*_Kur_) and the acetylcholine-activated inward rectifier potassium current (*I*_K, Ach_).^8^ However, the contribution of repolarizing potassium currents non-selectively expressed in atrium and ventricle may also be relevant in hiPSC-aCM. Among these currents, the slowly activating delayed rectifier current (*I*_Ks_) is of special interest, since its contribution to repolarization is unknown not only in hiPSC-aCM but also in the human atrium.^9^ Therefore, we have measured *I*_Ks_ currents in human atrial cardiomyocyte (aCM) and in hiPSC-aCM. We used an *in silico* model to identify conditions where *I*_Ks_ could contribute to repolarization in the human atrium. The findings were validated by AP measurements in intact atrial tissue and atrial engineered heart tissue (aEHT).

## Methods

### Human adult atrial tissue

Heart tissue from right atrial appendages was obtained with informed consent from 53 patients undergoing cardiac surgery at the Department of Heart Surgery, Dresden University of Technology. Work with human tissue was approved by the Medical Faculty Ethics Committee of Dresden University of Technology (document EK790799). Patch clamp experiments in human atrial cardiomyocytes and AP recordings in intact human atrial tissue were performed at the Department of Experimental Pharmacology and Toxicology, Medical Faculty, Dresden University of Technology, between 2004 and 2011. While patch clamp data were not used before from human atrial cardiomyocytes, basal AP data (before adding potassium channel blockers) were used for a larger retrospective analysis focusing on impact of sex and age on AP characteristics.^10^ Patients in AF were older than in SR, and ejection fraction was not reduced in both groups (57 and 51% in SR and AF, respectively). The majority of patients was treated with β-blockers, diuretics, and angiotensin-converting enzyme-inhibitors (for details, see *Table 1*).

**Table 1:** Patient characteristics

Patient characteristics	SR	AF
*N*	37	16
Gender (m/f)	26/11	13/3
Age (years)	65.2 ± 3.0	71.2 ± 1.9
BMI (kg/m^2^)	28.5 ± 2.5	29.4 ± 0.9
Hypertension, *n*	34	14
Diabetes mellitus, *n*	16	5
Hyperlipidaemia, *n*	33	4
CAD, *n*	22	0
VD, *n*	10	7
CAD+VD, *n*	5	9
LVEF (%)	56.9 ± 4.7	51.3 ± 3.6
LA (mm)	41.4 ± 2.1	54.0 ± 1.3
LVEDD (mm)	52.0 ± 2.4	52.0 ± 1.2
Cardiovascular medication (*n*)		
Digitalis glycosides	0	2
ACE-inhibitors	19	10
AT1-blockers	9	5
β-blockers	34	14
Ca^2+^ channel blockers	6	5
Diuretics	21	12
Nitrates	6	2
Lipid-lowering drugs	27	6

Mean ± SEM.

SR, sinus rhythm; AF, atrial fibrillation; CAD, coronary artery disease; VD, valve disease; LVEF (%), left ventricular ejection fraction; LA, left atrial diameter; LVEDD, left ventricular end-diastolic diameter; ACE, angiotensin-converting enzyme; AT_1_, angiotensin 1 receptor; VD, vascular diseases.

Human atrial cardiomyocytes (aCMs) were isolated and prepared as previously described.^11^ In brief, the tissues were cut into small pieces in Ca^2+^-free isolation buffer of the following composition (in millimolar): NaCl 100, KCl 10, KH_2_PO_4_ 1.2, MgSO_4_ 5, MOPS 5, glucose 20, and taurine 50 (pH 7.0). The buffer contained 30 mM butanedione monoxime (Sigma, St. Louis, MO, USA). During the entire isolation procedure, the solutions were oxygenated with 100% O_2_ at 37°C. Human aCMs were dissociated with protease type XXIV (5 U/mL; Sigma, St. Louis, MO, USA) and collagenase type 1 (254 U/mL; Worthington Biochemical, Lakewood, NJ, USA) for 15–35 min. Thereafter, the suspension was centrifuged at 80 g for 10 min, and the sediment or pellet was resuspended and stored until use in 4 mL Na^+^- and K^+^-free buffer of the following composition (in millimolar): KCl 20, H_2_PO_4_, taurine 10, L-glutamic acid 70, β-OH-butyrate 10, EGTA 10, albumin 1%, and glucose 10 (pH 7.4), supplemented with 0.5 mM Ca^2+^ in three steps at room temperature.

### Atrial differentiation of human induced pluripotent stem cell–derived atrial cardiomyocytes and generation of atrial engineered heart tissue

Work with hiPSC-CM conformed to the principles outlined by the Declaration of Helsinki. A skin fibroblast–derived hiPSC line was used for this study with informed consent of the donor. All procedures involving the generation and analysis of hiPSC lines were approved by the local ethics committee in Hamburg (Az PV4798). Procedures for hiPSC expansion, atrial cardiomyocyte differentiation, and EHT generation for hiPSC lines were performed according to published in-house standardized protocol.^12^ Shortly, as previously reported, embryoid bodies were generated from expanded hiPSC-stirred suspension using spinner flasks. Mesodermal induction was executed by a growth factor cocktail (2 ng/mL BMP-4 and BMP-5 ng/mL activin A: R&D Systems, Minneapolis, MN, USA, and Miltenyi Biotec, Cologne, Germany) for 3 days and the cardiac differentiation by WNT signal inhibitor XAV 939 (1 μM; Tocris, Wiesbaden-Nordenstadt, Germany). To induce differentiation towards a hiPSC-aCMs, RA (1 µM; Sigma, St. Louis) was added for the first 72 h of WNT signalling inhibition as recently described.^3,5,8^

EHTs were generated from 1 million hiPSC-aCMs per construct. The fibrin gel matrix was made by mixing hiPSC-aCM, fibrinogen (Sigma F4753, St. Louis, MO, USA), and thrombin (100 U/L, T7513, St. Louis, MO, USA), which were poured into agarose (1%) casting molds with silicone posts inserted from above.^12^ EHTs were cultured at 37°C in a humidified cell culture incubator with 7% CO_2_ and 40% O_2_ levels for 30 days before being used for experiments. The culture medium consisted of the following components: DMEM (Biochrom, Berlin, Germany), 10% heat-inactivated horse serum (Gibco, Paisley, Scotland), 1% penicillin–streptomycin (Gibco, Paisley, Scotland), insulin at a concentration of 10 µg/mL (Sigma, St. Louis, MO, USA), tranexamic acid at 400 μmol/L (Sigma-Aldrich 857653), and aprotinin at 33 µg/mL (Sigma, St. Louis, MO, USA). Dissociation of EHTs was performed^5,8^ with collagenase II (200 U/mL, 1 mg/mL, Worthington, NJ, USA) for 3.5 h at 37°C and dispersed by careful pipetting to receive hiPSC-aCMs. Isolated hiPSC-aCMs were plated on gelatin-coated (0.1%) glass coverslips (12 mm diameter; Carl Roth GmbH & Co, Karlsruhe, Germany). Subsequently, the cells were kept in culture for 24–48 h to maintain adherence under superfusion in the recording chamber during patch clamp measurements.

### Expression analysis by RNA sequencing

The expression analysis by RNA sequencing was performed by the Core Facility Genomics in Münster (Medical Faculty, Muenster, Germany). Total RNA was extracted from snap frozen EHTs using the RNeasy Mini Kit (Qiagen). High-quality total RNA (Agilent TapeStation 4200, RIN value check) was enriched for poly(A)-tailed RNA using the NEBNext Poly(A) magnetic isolation module (NEB) followed by NEBNext Ultra II directional RNA Library Preparation (NEB). Library was quality controlled (Agilent TapeStation 4200) and quantified (NEBNext Library Quant for Illumina, NEB), and an equimolar pool was sequenced in a single read mode, with 72 cycles on a NextSeq2000 system (Illumina).

### Current measurements

Ion currents were measured at 37°C in the whole-cell configuration using an Axopatch 200B amplifier (Axon Instruments, Foster City, CA, USA). The ISO2 software (MFK, Niedernhausen, Germany) was used for data acquisition and analysis. Heat-polished pipettes were pulled from borosilicate-filamented glass with an external diameter of 1.5 mm and internal diameter of 0.87 (HILG1103227; Hilgenberg, Malsfeld, Germany) with a DPZ-Universal puller (Zeitz Instruments, Munich, Germany). Tip resistances were 2.5–5 MΩ, and seal resistances were 3–6 GΩ. Cell capacitance (*C*_m_) was calculated from steady-state current during depolarizing ramp pulses (1 V/1 s) from −40 to −35 mV. The cells were investigated in a small perfusion chamber placed on the stage of an inverse microscope. Outward currents were measured with the following bath solution (in millimolar): NaCl 120, KCl 5.4, HEPES 10, CaCl_2_ 2, MgCl_2_ 1, and glucose 10 (pH 7.4, adjusted with NaOH). The internal solution included (in millimolar) the following: DL-Aspartate potassium salt 80, KCl 40, NaCl 8, HEPES 10, Mg-ATP 5, Tris-GTP 0.1, EGTA 5, and CaCl_2_ 2 (pH 7.4, adjusted with KOH). Currents were elicited by 5 s long test pulses to +50 mV, applied every 10 s (holding potential was kept at −40 mV to inactivate Na^+^ currents). Contaminating Ca^2+^ currents were suppressed with the selective L-type calcium channel blocker nisoldipine (10 µM; Sigma, PHR1290, St. Louis, MO, USA). Cells were exposed to a single concentration of the selective *I*_Ks_ blocker HMR-1556 (1 µM, Aventis Pharma, Germany).^13^ In some experiments, cells were exposed first to the non-selective β-adrenoceptor agonist (-)-isoprenaline (1 µM; Sigma, I5627, Sigma, St. Louis, MO, USA) and on top to HMR-1556. Bath solutions containing isoprenaline were freshly prepared every day from frozen stock solutions that were stored at −20°C.

*I*_Ks_ currents were calculated as difference traces subtracted from traces recorded in the absence and presence of HMR-1556. From 1 µM HMR-1556, we assumed a complete block of *I*_Ks_. We ignored the marginal block of other currents 1 µM HMR-1556 on (*I*_Ca, L_ ∼10% and *I*_to_ ∼5%). HMR-1556 at 1 µM does not block *I*_Kr_ in guinea pig ventricular cardiomyocytes.^13^ We defined a cell as having *I*_Ks_ by a persistent 10% drop in outward current upon 1 µM HMR-1556 within 1 min. Voltage dependency of *I*_Ks_ activation was estimated from HMR-1556–sensitive tail currents (at −40 mV) following different test pulse potentials. Currents were normalized to their maximum and plotted against the actual test pulse potential. A Boltzmann function was fitted to the normalized values by the following equation: *I*/*I*_0_ = 1 / (*1* + exp((*V*_m_ − *V*_0.5act_) / *k*_act_)), where *V*_m_, *V*_0.5act_, and *k*_act_ are the test pulse potential, the voltage of half-maximum activation, and the slope factor, respectively, using GraphPad Prism software version 6 (GraphPad Software, San Diego, CA, USA). Activation and deactivation kinetics were estimated by fitting single exponential functions to HMR-1556–sensitive difference traces with the ANA-3 software (MFK, Niedernhausen, Germany) by the following equation:


$$I_{m}=A*\exp(t/\tau )+B$$

where *A* is the amplitude, *τ* is the time constant of the exponentially increasing outward current or decreasing tail current *I*_m_, and *B* is the amplitude of the non-activating current component.

### Action potential measurement

AP measurements in intact atrial trabeculae and aEHT were performed with standard sharp microelectrode pulled from the glass capillaries mentioned above.^14^ Tip resistance was between 25 and 55 MΩ when filled with 2 M KCl. The aEHTs were transferred from the 24-well EHT culture plate into the recording chamber and fixed with needles in an optimal position for AP recording. Tissues were continuously superfused with Tyrode’s solution (in millimolar): NaCl 127, KCl 5.4, MgCl_2_ 1.05, CaCl_2_ 1.8, glucose 10, NaHCO_3_ 22, and NaHPO_4_ 0.42, balanced with O_2_-CO_2_ (95:5) at 36°C (pH 7.4).

The signals were amplified by a BA-1s npi amplifier (npi electronic GmbH, Tamm, Germany) and recorded and analysed offline using the Lab-Chart software (version 8, AD Instruments Pty Ltd., Castle Hill NSW, Australia). To block the rapidly activating delayed rectifier potassium current (*I*_Kr_), we used E-4031 (1 µM; 1808, Tocris Bioscience, UK) to block *I*_Kur_ 4-aminopyridine (4-AP) (100 µM; Sigma, St. Louis, MO, USA) and *I*_Ks_ HMR-1556 (1 µM; Aventis Pharma, Germany).

Drug effects were measured 15 min after addition of the drug, and to calculate the AP parameters, data from 15 consecutive APs were averaged. APD in aEHT was not corrected for rate.

### Mathematical modelling and computer simulations of *I*_Ks_ and action potential in human atrial cardiomyocytes

To analyse the contribution of *I*_Ks_ on repolarization in the human atrium, we used the 2016 version of our established computer model of the atrial cardiomyocyte.^15–17^ As previously published, mathematical formulations of *I*_Ks_ kinetics in human CMs have taken rather different approaches. In this case, we compared the *I*_Ks_ amplitude and dynamics *in silico* by using three previously published current formulations^18–20^ and a reparametrized formulation in the simulations (see Supplementary material online, *Figures S2*–*S6*). *I*_Ks_ activation at different heart rates was simulated, using a dynamic restitution protocol modified from Koller *et al.*^21^ Starting from a dynamic pacing steady state (at basic cycle length (BCL) = 1500 ms), BCL was decreased in 50 ms steps (BCL = 1500: −50:250), which corresponds to beating per minutes increasing from 40 to 240. At each BCL, 50 stimuli were applied, and the last AP for each BCL was saved for analysis. We adjusted the conductances of *I*_Kr_ and *I*_Kur_ in the human CM model slightly to improve the spike and dome AP shape and to better match the current block effects on AP morphology observed *in vitro*.

To model the effect of persistent AF *in silico*, we used our previously published scheme^15,17^ that accounts for the remodelling sarcolemmal ion currents, intracellular calcium handling, and cellular hypertrophy. We updated the *I*_Ks_ multiplication parameter from 2.45 to 1.97 to match the AF-related change in *I*_Ks_ that was shown in our new *in vitro* data.

Inhibitory effects of HMR-1556 and 4-AP on *I*_CaL_, *I*_to_, *I*_Kur_, *I*_Kr_, and *I*_Ks_ were accounted for in the simulations by using the standard single pore block model. Full details of the mathematical modelling and simulation approach are described in the Supplementary material online.

## Results

### Human induced pluripotent stem cell–derived atrial cardiomyocytes express genes encoding for Ks channel proteins and regulatory subunits

In the human heart, mRNA for KCNQ1, MinK1, KCNE1, and AKAP9^22^ is expressed in both atrium and ventricles.^23^ Thus, we measured if this holds true for ventricular and atrial EHTs generated from hiPSC-CM and hiPSC-aCM, respectively. While KCNQ1 mRNA abundance was significantly larger, AKAP9 was smaller in atrial than ventricular EHTs. Furthermore, KCNE1 and MinK1 mRNA abundance did not differ between atrial and ventricular EHTs (*Table 2*; Supplementary material online, *Figure S7*).

**Table 2:** Expression Ks channel proteins and regulatory subunits in atrial/ventricular EHT

Gene	Ventricular EHT	Atrial EHT
KCNQ1	1604 ± 95	2570 ± 93*
MINK1	1067 ± 69	1076 ± 84
KCNE1	28 ± 4	54 ± 9
AKAP9 (Yotiao)	2185 ± 130	1551 ± 130*

Summary of gene expressions in ventricular (*n* = 12/12) and atrial EHT (*n* = 3/3). Mean ± SEM; *n*/*n* indicates number of EHT/number of batches.

^*^ *P*-value < 0.05 ventricular vs. atrial EHT groups (paired test following ANOVA).

### Only a fraction of atrial cardiomyocytes possessed measurable *I*_Ks_, but frequencies and current amplitudes did not differ between sinus rhythm atrial cardiomyocytes, atrial fibrillation atrial cardiomyocytes, and human induced pluripotent stem cell–derived atrial cardiomyocytes

**Figure 1:**
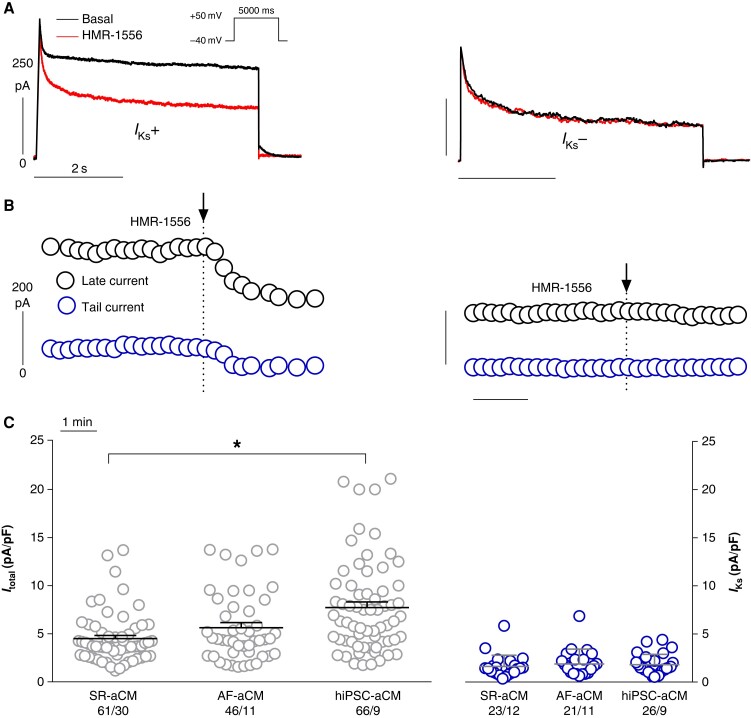
Identification of *I*_Ks_ by HMR-1556 and total outward currents and *I*_Ks_ in adult SR-aCM, AF-aCM, and hiPSC-aCM. (*A* and *B*) Superimposed original traces of currents before and after exposure to 1 µM HMR-1556 in a hiPSC-aCM with (left) and without (right) effect. Pulse protocol given as inset. Respective time courses of the current at the end of the test pulse to +50 mV (black, top) and tail currents at −40 mV (blue, bottom) below. Start of exposure to HMR-1556 indicated by arrows. (*C*) left: Outward current densities in individual adult SR-aCM, AF-aCM, and hiPSC-aCM and measured at the end of the 5 s long test pulse to +50 mV in the absence of HMR-1556. *P < 0.05, unpaired *t*-test, mean ± SEM; number indicates number of cells/patients or cells/EHT. Right: Current densities of *I*_Ks_ (defined as HMR-1556–sensitive current) for adult SR-aCM, AF-aCM, and hiPSC-aCM.

Outward currents in aCM did not show a slowly activating component as observed in human ventricular CM.^9^ However, some cells showed deactivating tail currents when jumping back to −40 mV (*Figure 1A* basal, left). To unmask the potential contribution of *I*_Ks_ to the outward current, we used the selective *I*_Ks_ blocker HMR-1556. An abrupt persistent 10% drop in outward current upon HMR-1556 was used to define the existence of *I*_Ks_ (*Figure 1A*, left). Only 23 out of 61 aCMs from patients in SR possessed *I*_Ks_ (37.7%). The frequency was not statistically different between aCMs to that of patients in AF (21 of 46; 45.7%) or hiPSC-aCM (26 of 66; 39.4%; *Table 3*). Tail currents were completely blocked by HMR-1556 (*Figure 1A* and *B*). Outward currents before adding HMR-1556 were significantly larger in hiPSC-aCM than in aCM from SR and AF. In contrast, current densities for *I*_Ks_ did not differ between hiPSC-aCM and aCMs from SR or AF (*Figure 1C*, right, and *Table 3*).

**Table 3:** *I*_Ks_ characteristics in aCM from patients with sinus rhythm (SR) and atrial fibrillation (AF) and in hiPSC-aCM

	SR-aCM	AF-aCM	hiPSC-aCM
Capacitance pF	73.8 ± 2.9 61/30	94.6 ± 5.2* 46/11	23.2 ± 1.5* 66/9
*I*_Ks_ present (%)	37.7	45.7	39.4
*I*_total_ @+50 mV (pA)	314.7 ± 20.7 61/30	499.0 ± 46.8* 46/11	187.1 ± 20.8* 66/9
*I*_total_ density @+50 mV (pA/pF)	4.5 ± 0.3 61/30	5.9 ± 0.7 46/11	7.7 ± 0.6* 66/9
*I*_Ks_ @+50 mV (pA)	115.0 ± 15.4 23/12	199.3 ± 45.4 21/11	48.8 ± 7.8* 26/9
*I*_Ks_ density @+50 mV (pA/pF)	1.6 ± 0.2 23/12	1.9 ± 0.3 21/11	1.8 ± 0.2 26/9
*I*_Ks_ tail current density @−40 mV (pA/pF)	0.3 ± 0.0 5/5	0.5 ± 0.0 8/2	0.7 ± 0.07 32/5
*V*_act0.5_ (mV)	0.0 ± 2.3 5/5	7.4 ± 2.3 8/2	6.0 ± 2.3 4/1
*t*_activation_ @ +50 mV (ms)	180.9 ± 37.7 7/5	185.7 ± 19.9 6/2	182.5 ± 16.2 9/3
*t*_deactivation_ @ −40 mV (ms)	88.1 ± 0.7 5/5	88.9 ± 3.0 8/4	109.5 ± 5.8 5/2

Mean ± SEM; number indicates number of cells/patients or cells/EHT.

^*^ *P* < 0.05, unpaired *t*-test compared with SR-aCM and AF-aCM or hiPSC-aCM.

### *I*_Ks_ did not respond to β-adrenoceptor stimulation in atrial cardiomyocytes or in human induced pluripotent stem cell–derived atrial cardiomyocytes

There is a substantial increase in *I*_Ks_ (three- to four-fold) upon β-adrenoceptor (β-AR) stimulation in dog ventricular CM.^24^ Thus, we wanted to evaluate whether *I*_Ks_ is activated by β-AR stimulation also in human aCM and in hiPSC-aCM. Cells were first exposed to the non-selective β-AR agonist isoprenaline (1 µM) and subsequently, in the continuous presence of isoprenaline, to HMR-1556. Total outward currents did not increase after adding isoprenaline (see Supplementary material online, *Figure S1*) even in cells that showed clear *I*_Ks_, as defined by a drop in outward current upon subsequent exposure to HMR-1556. The fraction of ‘responders’ to HMR-1556 did not differ between cells pre-treated or not pre-treated with isoprenaline. Furthermore, the effect size of HMR-1556 in ‘responders’ was not larger in isoprenaline-treated cells compared with those not treated in SR-aCM and in hiPSC-aCM (in SR: 1.5 ± 0.2 pA/pF *n* = 16/2 vs. 1.6 ± 0.2 pA/pF *n* = 23/12; in hiPSC-aCM: 2.1 ± 0.5 pA/pF *n* = 7/3 vs. 1.8 ± 0.2 pA/pF *n* = 26/9).

### *I*_Ks_ in human induced pluripotent stem cell–derived atrial cardiomyocytes showed voltage and time dependency of activation as seen in adult atrial cardiomyocytes

**Figure 2:**
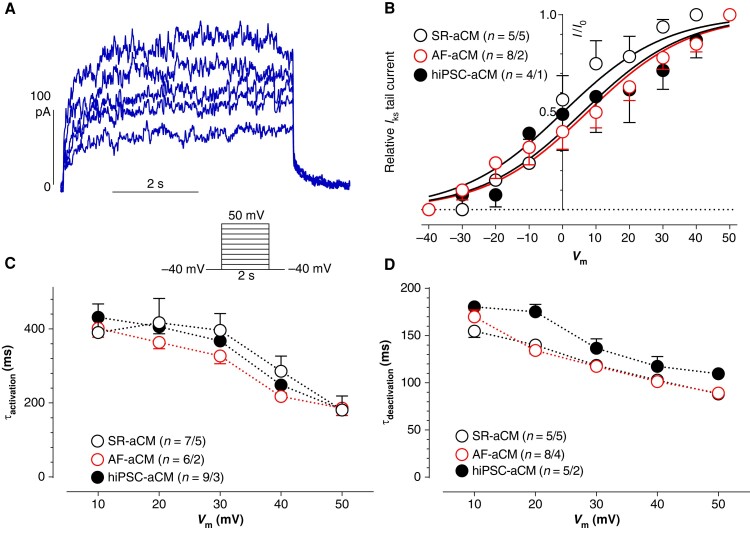
Time (activation and deactivation) and voltage dependency of *I*_Ks_ in adult SR-aCM, AF-aCM, and hiPSC-aCM. (*A*) Original traces of HMR-1156–sensitive currents present slow activation and deactivation properties. Plus protocol is given inset. (*B*) The amplitude of *I*_Ks_ tail current was measured upon return to a holding potential of −40 mV after 5 s voltage step to different test pulse potentials. Amplitudes were normalized to its maximum (*I*/*I*_0_). Mean values ± SEM: numbers in brackets indicate cells/patients or cells/EHT. (*C* and *D*) The activation and deactivation kinetics at increasing test pluses. Mean values ± SEM: numbers in brackets indicate cells/patients or cells/EHT.

The contribution of *I*_Ks_ to repolarization depends on voltage and time dependency of activation. The voltage dependency of *I*_Ks_ activation was estimated from HMR-1556–sensitive tail currents at −40 mV following different test pulse potentials (*Figure 2A*). The voltages to achieve half-maximum activation (*V*_act0.5_) did not differ between aCMs from SR, AF, and hiPSC-aCM (0, 7, and 6 mV, respectively; *Figure 2B* and *Table 3*).

The contribution of a time-dependent activating current to the AP shape depends on its activation and deactivation kinetics. Therefore, we fitted mono-exponential functions to HMR-1556–sensitive current traces recorded at different test pulse potentials to estimate the time course of activation kinetics over a larger voltage range (*Figure 2C* and *D*). The respective tail currents were used to measure deactivation kinetics. Both activation and deactivation of *I*_Ks_ were faster at more positive test pulse potentials, but again, time constants did not differ between SR-aCM, AF-aCM, and hiPSC-aCM (*Figure 2C* and *D* and *Table 3*).

### Reparameterized mathematical formulation to recapitulate *I*_Ks_ activation and deactivation dynamics

**Figure 3:**
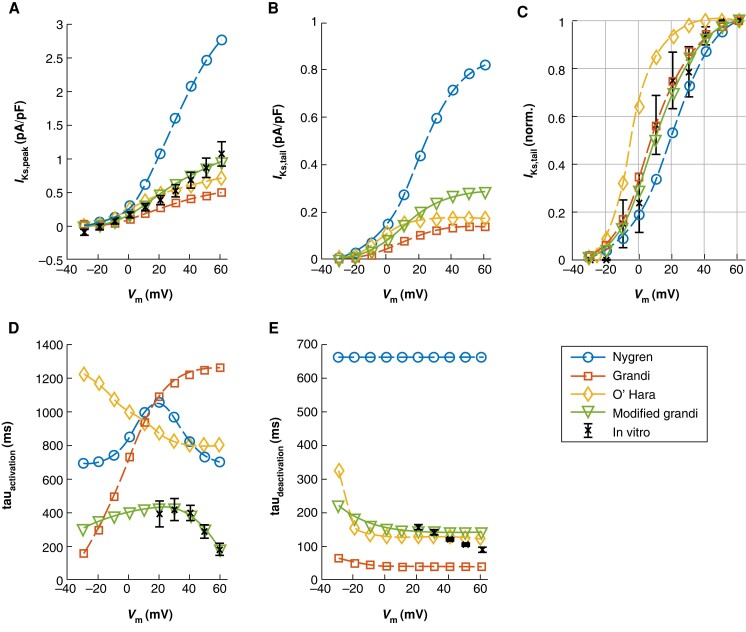
Computer model of the slow delayed rectifier potassium current reparameterized to match the characteristics measured *in vitro*. The simulation results obtained with new current formulation are compared with the previous mathematical formulations: Nygren, Nygren *et al.* (1998); Grandi, Grandi *et al.* (2010); and O’Hara, O’Hara *et al.* (2011). (*A*) Peak current amplitude, (*B*) tail current amplitude, and (*C*) normalized tail current amplitude as a function of voltage. (*D* and *E*) Time constants of activation and deactivation of the currents.

To investigate to what extent *I*_Ks_ can contribute to atrial repolarization, we used our established computer model. While the *I*_Ks_ formulations developed previously for a human CM model have voltage dependencies resembling the *in vitro* results with reasonable accuracy, none of them could replicate the activation and deactivation kinetics of the current we saw in aCM (*Figure 3*). We chose the formulation by Grandi *et al.*^18^ since in this model, the voltage dependency needed to be only slightly tuned, as a starting point for a reparameterized *I*_Ks_ formulation. After reparameterization, the modified *I*_Ks_ had activation and deactivation kinetics closely matching the *in vitro* findings (*Figure 3D* and *E*). The properties of the four *I*_Ks_ formulations as well as the raw *in silico* data analysed in *Figure 3* are shown in Supplementary material online, *Figures S2* and *S3*, respectively. Importantly, when we used an alternative voltage clamp protocol to validate the modified model, it matched better with the *in vitro* data from Virág *et al.*^25^ than any of the previously published *I*_Ks_ formulations (see Supplementary material online, *Figure S4*).

**Figure 4:**
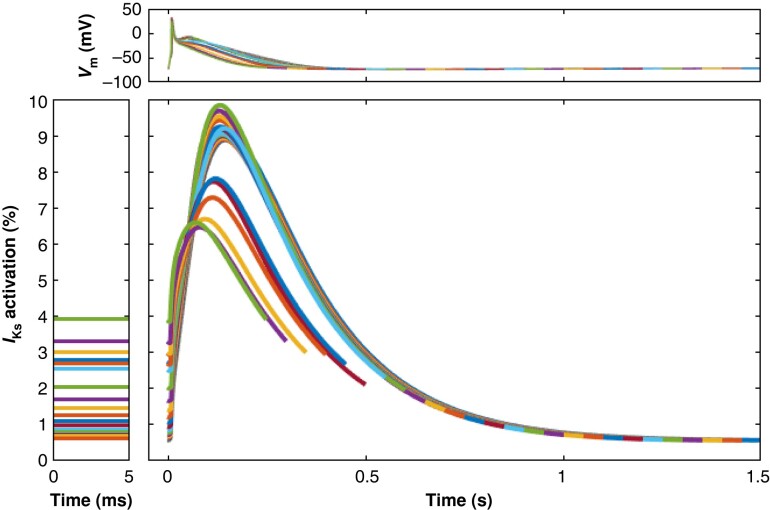
No evidence for *I*_Ks_ accumulation in the human atrium at fast beating rate. Simulated *I*_Ks_ activation during AP at different cycle lengths (from 1500 to 250 ms). Top: Action potentials used for dynamic clamping are given on. Bottom: *I*_Ks_ activation is given as per cent of maximum activation. Center: Activation of *I*_Ks_ during AP. Left: Residual *I*_Ks_ activation at the end of the diastole. Note: There is some residual activation of *I*_Ks_ at higher rate left panel, showing *I*_Ks_ activation at the end of the diastolic potential. However, maximum activation during AP decreases.

Next, we performed a computer simulation to test whether *I*_Ks_ accumulation can occur at high fast beating rate under our conditions. While the ‘residual activation’ does increase (zoomed bottom left panel of *Figure 4*), the gradual decrease of APD_50_ and drop of the plateau voltage to more negative values more than compensate that effect. Thus, peak *I*_Ks_ activation is actually decreasing with increasing pacing frequency (center panel of *Figure 4*).

### *In silico* model predicts only a small contribution of *I*_Ks_ on repolarization in the human atrium even when repolarization reserve is reduced

**Figure 5:**
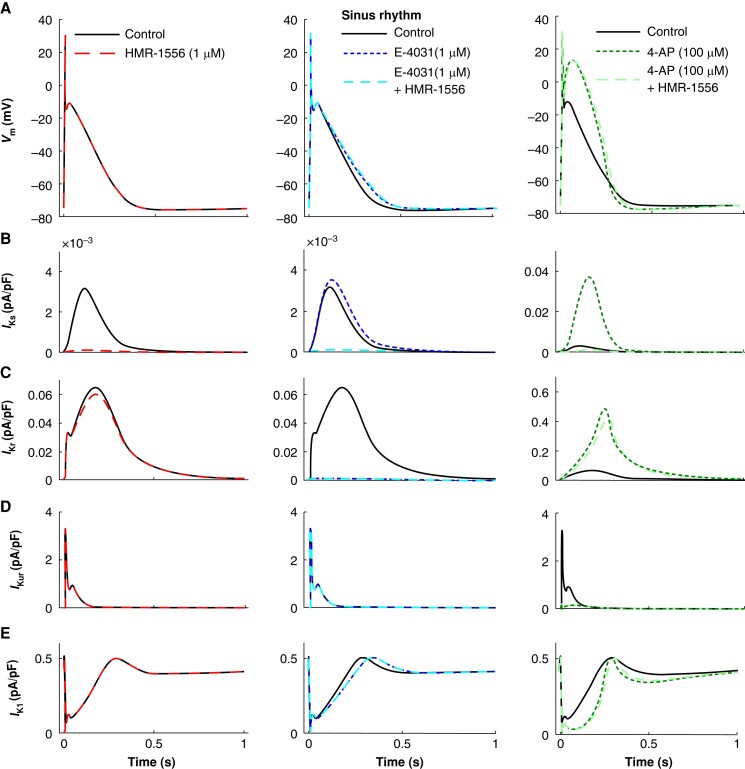
Simulated drug effects in sinus rhythm variant of the computational cell model. (*A*) Impact of HMR-1556 (left), E-4031, E-4031 + HMR-1556 (middle), and 4-AP and 4-AP + HMR-1556 (right) on the action potential morphology. HMR-1556 application increased APD_90_ by a mere 1.9%, whereas E-4031 increased it by 16.7% and the combination by 18.2%. 4-AP increased APD_90_ by −2.5% and the combination by 4.3%. (*B*–*E*) Underlying changes in the slow, rapid, and ultra-rapid activating delayed rectifier potassium currents and the inward rectifier potassium current.

**Figure 6:**
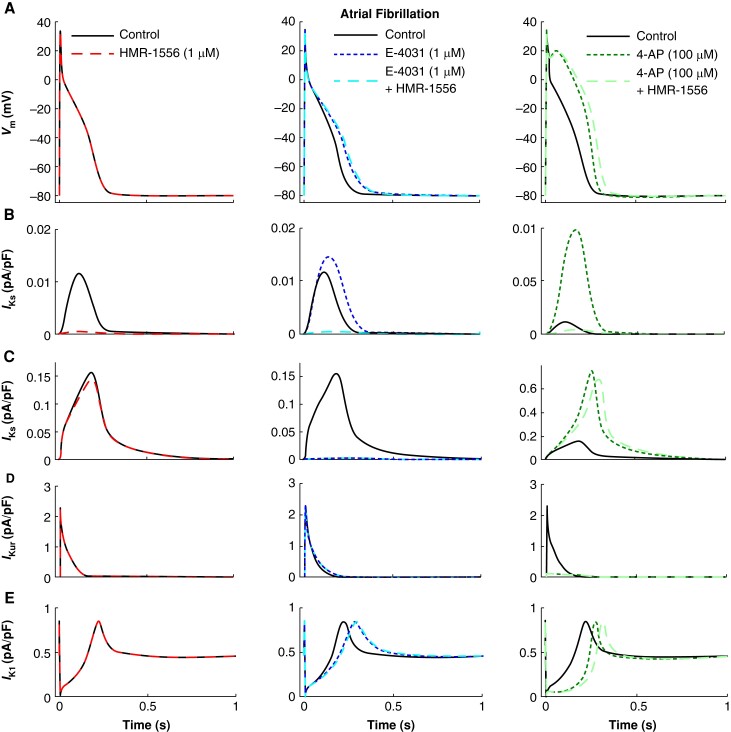
Simulated drug effects in the atrial fibrillation variant of the computational cell model. (*A*) Impact of HMR-1556 (left), E-4031, E-4031 + HMR-1556 (middle), and 4-AP and 4-AP + HMR-1556 (right) on the action potential morphology. HMR-1556 application increased APD90 by 2.3%, whereas E-4031 increased it by 30.5% and the combination by 33.3%. 4-AP increased APD90 by 21.3% and the combination by 38.5%. (*B*–*E*) Underlying changes in the slow, rapid, and ultra-rapid activating delayed rectifier potassium currents and the inward rectifier potassium current.

In order to estimate whether the reparameterized *I*_Ks_ can contribute to repolarization in human aCM, we used an *in silico* model (*Figures 5* and *6*). The model predicted activation of *I*_Kr_ and *I*_Ks_ during the AP plateau phase. Computer modelling revealed that in SR under basal conditions, *I*_Kr_ maximum current density was 20-fold larger than for *I*_Ks_ (*Figure 5B* and *C*). Consequently, block of *I*_Kr_ with E-4031 increased APD_90_ substantially by 16.7%, while block of *I*_Ks_ by HMR-1556 had a negligible impact (1.5%; *Figure 5A*, center; Supplementary material online, *Table S2*). E-4031 did not prolong APD_20_. Slowing of repolarization started in the plateau phase, thereby increasing APD_50_ (see Supplementary material online, *Table S3*). Plateau voltage remained unaffected. As a result of *I*_Kr_ block, *I*_Ks_ activation increased by about 16% (*Figure 5B*, center). However, even on top of E-4031, the predicted increase in APD_90_ by HMR-1556 remained marginal (1.9% vs. 1.5%, *Figure 5A*, left, in the absence of E-4031 vs. center in the presence of E-4031; Supplementary material online, *Table S2*).

Block of *I*_Kur_ with 4-AP in SR shifted the plateau of the AP to positive voltages (*Figure 5A*, right) and therefore increased substantially activation of both *I*_Ks_ and *I*_Kr_ (13-fold for *I*_Ks_ and 8-fold for *I*_Kr_; *Figure 5B* and *C*, right). However, the maximum amplitude of *I*_Kr_ was still 10-fold higher than that of *I*_Ks_ (*Figure 5C* vs. *B*, right). Thus, even in the presence of *I*_Kur_ block, the predicted prolongation APD_90_ by HMR-1556 remained negligible (*Figure 5A*, right; Supplementary material online, *Table S2*).

Next, we simulated *I*_Ks_ contribution to repolarization in AF. Our computer model nicely reproduced the higher plateau potential in AF than in SR^10^ under basal conditions (*Figure 5A* vs. *6A*), leading to a stronger activation of both *I*_Ks_ and *I*_Kr_ compared with SR (four-fold vs. two-fold; *Figure 5B* and *C*, left, vs. *Figure 6B* and *C*, left). Again, as seen in SR, maximum activation of *I*_Kr_ was still more than 10 times higher than that of *I*_Ks_ (*Figure 6B* vs. *C*). Consequently, *I*_Kr_ but not *I*_Ks_ dominated repolarization, since predicted prolongation of APD_90_ by block of *I*_Kr_ was 30.5%, while prolongation by *I*_Ks_ block was marginal (+2.3%; *Figure 6A*, center; Supplementary material online, *Table S2*). *I*_Kr_ block slightly prolonged the plateau phase (see Supplementary material online, *Table S3*) that led to a stronger activation of *I*_Ks_ (*Figure 6B*). However, the maximum activated *I*_Ks_ was still very small (*Figure 6B*, center). Thus, even in the presence of E-4031, application of HMR-1556 prolonged APD_90_ by only +2.8% (*Figure 6B*, center; Supplementary material online, *Table S2*).

Block of *I*_Kur_ in AF almost doubled APD_20_ (*Figure 6A*, right), increasing activation of both *I*_Ks_ and *I*_Kr_ (eight-fold for *I*_Ks_ and five-fold for *I*_Kr_; *Figure 6B* and *C*, right). Since activation of both *I*_Kr_ and *I*_Ks_ was larger in AF even under basal conditions (in the absence of *I*_Kur_ block), maximum *I*_Ks_ amplitudes reached now values close to 0.1 pA/pF, now 30 times higher than in SR (basal in *Figure 5B*). In contrast, *I*_Kr_ amplitudes were 10-fold larger in AF (*I*_Kur_ blocked; *Figure 6C*, right) compared with SR (basal; *Figure 5C*, left). As a result, repolarization is dominated by *I*_Kr_ vs. *I*_Ks_ to a lesser extent facilitating contribution of *I*_Ks_ to repolarization resulting in a predicted increase in APD_90_ by 15% (*Figure 6A*; Supplementary material online, *Table S2*).

### Model validation by *in vitro* measurements of action potential in human atrial tissue

Models are an attractive tool to study cardiac electrophysiology. Utility of a model depends on how closely such a model recapitulates real findings. Therefore, we have compared the *in silico* predictions on *I*_Ks_ contribution to repolarization to *in vitro* findings obtained from human atrial trabeculae (both SR and AF).

**Figure 7:**
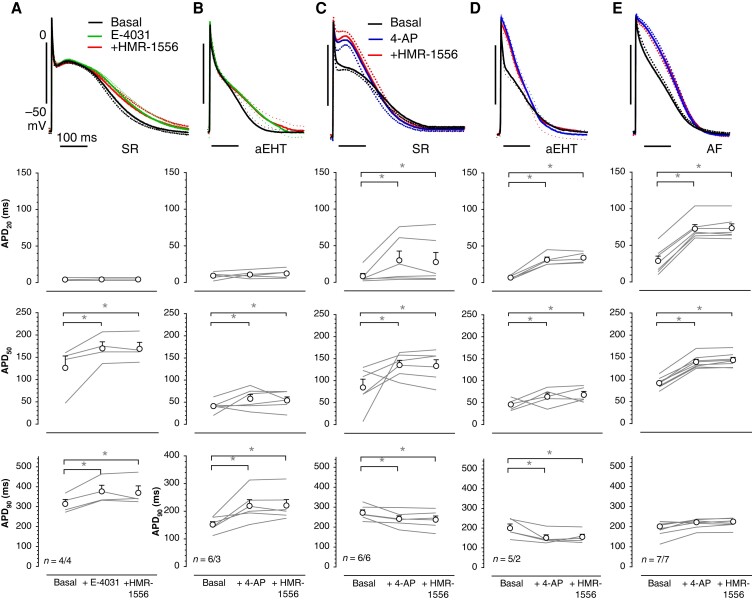
Reduction of repolarization reserve by block of ultra-rapid and rapid potassium outward currents did not unmask contribution of *I*_Ks_ to action potential shape in human adult atrial tissue and in aEHT. AP traces are presented as the average of all group experiments (solid lines) and SEM (dotted lines) in SR (*A* and *C*), in AF (*E*), and in atrial EHT (*B*, *D*). Control APs (black) and after 15 min of exposure to the respective blockers (100 µM 4-aminopyridine, 1 µM E-4031, and 1 µM HMR-1556). Graphs below show individual data points and mean ± SEM. Numbers indicate tissues/patients or EHT/batch.

In preliminary experiments, AP shape in atrial trabeculae from both SR and AF did not change upon exposure to HMR-1556 alone (data not shown). Therefore, we tested whether model predictions on *I*_Ks_ contribution under reduced repolarization reserve are correct. Firstly, we blocked *I*_Kr_ with E-4031 (1 µM). In SR, E-4031 prolonged APD_90_, while APD_20_ remained unchanged. HMR-1556 on top of E-4031 did not prolong APD_90_ further (*Figure 7A* and *Table 4*). In a next set of experiments, we blocked *I*_Kur_ first. For this purpose, we exposed tissues to 100 µM 4-AP and subsequently in the continuous presence of 4-AP to HMR-1556 (1 µM). As seen before in SR,^26^ 4-AP increased APD_20_ and shifted plateau voltage to positive values but shortened APD_90_. Importantly, in the context of the present study, HMR-1556 on top of 4-AP did not prolong APD_90_ (*Figure 7C* and *Table 5*).

**Table 4:** Effect of concomitant block of *I*_Kr_ and *I*_Ks_ in SR, AF, and aEHT

	Basal	+E-4031	+HMR-1556
SR (*n* = 4/4)			
RMP (mV)	−73.3 ± 1.7	−73.3 ± 1.6	−72.3 ± 2.2
APA (mV)	94.7 ± 2.3	93.0 ± 2.1	91.3 ± 2.2
*V*_max_ (V/s)	231.6 ± 17.9	226.0 ± 33.1	231.0 ± 28.2
APD_20_ (ms)	4.3 ± 0.9	4.5 ± 0.9	4.5 ± 0.9
APD_50_ (ms)	126.3 ± 26.6	170.0 ± 14.8*	169.0 ± 14.6
APD_90_ (ms)	313.8 ± 22.2	376.5 ± 30.9*	369.3 ± 34.8
*V*_Plateau_ (mV)	−15.3 ± 1.5	−14.8 ± 1.0	−16.8 ± 1.3
aEHT (*n* = 6/2)			
MDP (mV)	−70.5 ± 4.1	−65.9 ± 3.8	−66.4 ± 5.1
APA (mV)	90.2 ± 3.9	85.3 ± 8.4	89.8 ± 11.7
*V*_max_ (V/s)	147.2 ± 26.3	147.2 ± 20.6	115.4 ± 22.7
APD_20_ (ms)	9.3 ± 1.8	10.8 ± 1.7	12.4 ± 2.7
APD_50_ (ms)	41.2 ± 5.4	57.8 ± 9.3*	54.0 ± 8.1
APD_90_ (ms)	152.0 ± 10.5	219.5 ± 22.2*	220.8 ± 21.3
*V*_Plateau_ (mV)	−26.3 ± 2.8	−26.2 ± 2.9	−26.8 ± 1.1

Summary of action potential characteristics under basal conditions, in the presence of 1 µM E-4031 and in the concomitant presence of 1 µM E-4031 + 1 µM HMR-1556. Parameters are resting membrane potential (RMP) for SR; maximum diastolic potential (MDP) for EHT; amplitude of action potential (APA); maximum upstroke velocity (*V*_max_); action potential duration at 90%, 50%, and 20% repolarization (APD_20_, APD_50_, and APD_90_); and plateau (*V*_Plateau_). Mean ± SEM; n/*n* indicates number of tissues/number of patients or number of EHT/number of batches in case of atrial EHTs.

^*^ *P*-value < 0.05 basal vs. E-4031 groups (paired test following ANOVA).

**Table 5:** Effect of concomitant block of *I*_Kur_ and *I*_Ks_ in SR, AF, and aEHT

	Basal	+4-AP	+HMR-1556
SR *n* = 6/6			
RMP (mV)	−70.3 ± 1.2	−71.3 ± 1.5	−71.2 ± 1.8
APA (mV)	92.7 ± 3.0	93.2 ± 4.5	91.7 ± 4.8
*V*_max_ (V/s)	161.5 ± 25.4	156.0 ± 30.4	133.8 ± 23.8
APD_20_ (ms)	8.3 ± 3.8	30.0 ± 12.7*	27.7 ± 13.1
APD_50_ (ms)	84.5 ± 18.8	135.5 ± 10.7*	133.3 ± 14.1
APD_90_ (ms)	272.0 ± 14.5	241.2 ± 15.5*	237.2 ± 18.4
*V*_Plateau_ (mV)	−18.7 ± 1.7	3.8 ± 4.0*	2.4 ± 3.6
aEHT *n* = 5/2			
MDP (mV)	−74.2 ± 2.1	−72.3 ± 4.0	−67.8 ± 5.5
APA (mV)	95.8 ± 9.4	93.0 ± 9.9	93.8 ± 10.4
*V*_max_ (V/s)	148.1 ± 28.9	100.0 ± 24.9	98.3 ± 23.9
APD_20_ (ms)	6.8 ± 1.6	31.2 ± 3.6*	34.0 ± 3.2
APD_50_ (ms)	45.8 ± 5.7	63.6 ± 8.4*	67.8 ± 7.7
APD_90_ (ms)	200.0 ± 20.6	151.0 ± 15.1*	155.4 ± 13.8
*V*_Plateau_ (mV)	−31.0 ± 2.5	−8.8 ± 3.6*	−3.2 ± 3.5
AF (*n* = 7/7)			
RMP (mV)	−76.5 ± 1.1	−75.6 ± 0.9	−75.7 ± 1.1
APA (mV)	98.8 ± 1.5	102.2 ± 1.2	101.6 ± 1.1
*V*_max_ (V/s)	170.0 ± 15.4	164.0 ± 15.8	161.9 ± 12.5
APD_20_ (ms)	28.7 ± 6.5	72.7 ± 5.6*	73.6 ± 5.8
APD_50_ (ms)	91.7 ± 4.9	139.6 ± 6.0*	143.6 ± 6.2
APD_90_ (ms)	201.4 ± 8.5	221.0 ± 5.5	225.6 ± 5.1
*V*_Plateau_ (mV)	−6.2 ± 4.6	15.6 ± 1.5	14.5 ± 1.8

Summary of AP characteristics under basal conditions, in the presence of 100 µM 4-aminopyrodine (4-AP) and in the concomitant presence of 100 µM 4-AP + 1 µM HMR-1556. Parameters are resting membrane potential (RMP) for SR and AF; maximum diastolic potential (MDP) for EHT; amplitude of action potential (APA); maximum upstroke velocity (*V*_max_); action potential duration at 90%, 50%, and 20% repolarization (APD_20_, APD_50_, and APD_90_); and plateau (*V*_Plateau_). Mean ± SEM; *n*/*n* indicates number of tissues/number of patients or number of EHT/number of batches in case of atrial EHTs.

^*^ *P*-value < 0.05 basal vs. 4-AP groups (paired test following ANOVA).

#### In silico model for atrial fibrillation

We could confirm key findings in AF: the basal APD_20_ values were much longer, *V*_PLT_ less negative, and APD_90_ much shorter than SR.^26^ As shown before, for a larger number of human right atrial tissues,^10^ APD_20_ was longer and plateau voltage was higher in AF compared with SR (*Figure 7C* and *E*; Supplementary material online, *Table S1*). In AF, 4-AP increased not only APD_20_ but also APD_90_. However, in contrast to model predictions, block of *I*_Ks_ on top of *I*_Kur_ block did not prolong APD_90_ further (*Figure 7E* and *Table 5*).

#### Atrial engineered heart tissue as a model for sinus rhythm

Here, we present physiological current densities and biophysical properties of *I*_Ks_ in hiPSC-aCM. In order to evaluate whether our findings on *I*_Ks_ in hiPSC-aCM translate to the negligible contribution of *I*_Ks_ to repolarization as seen in SR, we measured AP in aEHT.

Block of *I*_Ks_ with HMR-1556 alone did not prolong APD (data not shown) in aEHT. Block of *I*_Kur_ with 100 µM 4-AP prolonged APD_20_ but shortened APD_90_. HMR-1556 on top of 4-AP did not prolong APD (*Figure 7D* and *Table 5*). Furthermore, E-4031 did not prolong early repolarization phase (APD_20_) but also plateau phase (APD_50_) and late repolarization (APD_90_). Again, HMR-1556 was without effect on APD (*Figure 7B* and *Table 4*).

It should be noted that aEHT beats spontaneously at 3.1 ± 0.2 Hz. APD_90_ (169.1 ± 12.7 ms) in aEHT was similar to APD_90_ in atrial tissue (SR) when paced at 3 Hz.^27^ Importantly, APD_90_ but not APD_20_ did not shorten with higher pacing rate in human atrial tissue.^27^ Thus, APD_20_ in aEHT seems (at least at 3 Hz) was not different than in SR at 1 Hz (see Supplementary material online, *Table S1*).

aEHT may represent a nice model to study human electrophysiology, since they recapitulate lack of *I*_Ks_ contribution to repolarization even when other potassium currents in SR are blocked. *In silico* models for human atrial AP need further improvement to reproduce the physiological situation in SR. Slowly beating aEHTs are needed to investigate whether EHTs are useful to study electrical remodelling as seen in AF.

## Discussion

The main results of our study are as follows:

1. HMR-1556 revealed the presence of *I*_Ks_ in ∼half of human aCM.
2. The properties of *I*_Ks_ in hiPSC-aCM closely resemble the situation in aCM.
3. *I*_Ks_ does not contribute relevantly to AP shape in human atrial tissues in SR or in aEHT.

###  

#### Detection of *I*_Ks_ in human atrial cardiomyocytes

In ventricular CM from several species (human, guinea pig, and dog), potassium outward currents are dominated by the slowly activating *I*_Ks_.^9,24,28^ In contrast, in human aCM, large rapidly activating transient potassium outward currents sensitive to 4-AP^29^ mask *I*_Ks_. This is due to the fact that tau values for inactivation of transient potassium outward currents^30,31^ lie in the same range as those for activation of *I*_Ks_ (600 ms at +50 mV, 36°C). Consequently, early studies in human aCM have defined *I*_Ks_ as the current resistant to high concentrations of 4-AP (2 mM), which block the transient potassium outward currents in the human atrium: *I*_Kur_ and *I*_to_.^32–34^ With the development of HMR-1556, blocking *I*_Ks_ but not I_Kur_ and *I*_to_,^13^ direct proof for *I*_Ks_ became available.

Our findings that not all CMs possess *I*_Ks_ are in line with the findings in human ventricular CM, where *I*_Ks_ was defined by other *I*_Ks_ blockers (L-735,821 or chromanol 293B). The results are difficult to directly compare to our findings, because in both studies, the researchers used pipette solutions containing the direct adenylyl cyclase activator forskolin in order to facilitate detection of *I*_Ks_.^25,35^ However, even under these conditions, *I*_Ks_ was found in only ∼50% of human ventricular CM.^25^ No data are given for experiments without forskolin. In another study, *I*_Ks_ was found in only 1 out of 53 human aCMs. Unfortunately, the authors did not report the method to identify *I*_Ks_.^36^ In addition, there is only one paper on *I*_Ks_ in hiPSC-aCM, which reported HMR-1556–sensitive outward currents. However, current traces miss the typical slow activation and deactivation, questioning whether the HMR-1556–sensitive currents do in fact represent *I*_Ks_.^37^

#### Biophysics of *I*_Ks_ in human atrial cardiomyocytes

The current density and the speed of activation affect the contribution of an ion current to AP shape. Current densities for activating *I*_Ks_ at the end of the test pulse are not given for human ventricular CM.^25,35^ However, from tail current amplitudes in both aCMs (SR and AF), we can conclude that *I*_Ks_ tail currents (SR-aCM: 0.3 ± 0.0, *n* = 5/5; AF-aCM: 0.5 ± 0.0 at +50 mV) are similar in human ventricular CM (0.2 pA/pF at +50 mV^25^; 0.25 pA/pF at +30 mV^35^). However, in hiPSC-aCM, *I*_Ks_ tail currents (hiPSC-aCM 0.7 ± 0.0, *n* = 32/5 at +50 mV) were almost four times larger than that in human ventricular CM.

The voltage dependency of *I*_Ks_ activation in hiPSC-aCM and aCM (SR and AF) was close to values reported for human ventricular CM in one study^9^ (*V*_act0.5_ ∼0 vs. ∼+10 mV). In contrast, Pérez-Hernández et al^32^ reported much more positive values in human aCM (*V*_act0.5_ in SR +35 mV), whereas in AF, *V*_act0.5_ was substantially shifted to less positive values than in SR (+13 mV). It remains open whether different experimental conditions may account for the differences (room temperature vs. 37°C in our study). Fast heart beat can favour accumulation of *I*_Ks_ in dog ventricular cardiomyocytes.^38^ Our simulation results suggest that under conditions (AP shape, *I*_Ks_ kinetics as found here), there is no ‘accumulation’ of *I*_Ks_ activation at higher pacing frequencies in the human atrium.

Anyhow, we cannot confirm that AF remodelling would affect voltage dependency of *I*_Ks_ activation. However, there seems to be a clear effect of temperature on *I*_Ks_ kinetics. While deactivation kinetics measured in aCM and in hiPSC-aCM (120 ms) match nicely with data in human ventricular CM,^25,35^  *I*_Ks_ activation at room temperature was slower than at 37°C: tau was 1.8 s at room temperature^32^ vs. ∼400 ms at 37°C in our study. Furthermore, activation of *I*_Ks_ in human aCM and in hiPSC-aCM seems to be generally faster than in human ventricular CM (tau at +50 mV, 37°C: 800 ms^9^). At present, we cannot explain differences in *I*_Ks_ biophysics between human aCM (this study) and human ventricular CM.^25,35^ Our findings suggest potentially relevant chamber-dependent differences in *I*_Ks_ biophysics. Anyhow, our results clearly indicate that *I*_Ks_ is present in hiPSC-aCM and that *I*_Ks_ biophysics closely resembles the situation in human adult aCM.

#### Contribution of *I*_Ks_ to repolarization in the human atrium when repolarization reserve is reduced

Biophysical properties of *I*_Ks_ were measured by rectangular voltage step protocols with very positive test pulse potentials and very long test pulse durations. We have used such an approach to better compare our results to findings from the literature (see above). However, contribution of *I*_Ks_ to repolarization depends on membrane voltage during an AP. In SR, APD_20_ is very short and plateau voltage quite negative making *I*_Ks_ contribution to repolarization very unlikely. Using an AP as a command pulse for voltage clamping^39^ also called ‘dynamic clamping’ is a very elegant approach to detect activation of *I*_Ks_ (as a HMR-1556–induced current) under physiological conditions. Here, we used a different approach to determine whether *I*_Ks_ can be activated during an AP in the human atrium. Our *in silico* model demonstrates activation of *I*_Ks_ during an atrial AP in SR but to a much lesser extent than *I*_Kr_. Findings resemble the situation in dog ventricles, where *I*_Kr_ unmasks *I*_Ks_ contribution to repolarization.^9,40^ Situation is different in the human heart where block of *I*_Kr_ alone did not unmask *I*_Ks_ contribution in the ventricles^9^ and also not in the human atrium (SR and AF, this study). In the human atrium, early repolarization is dominated by a potassium current that is not present in the ventricles: *I*_Kur_. Block of *I*_Kur_ can markedly increase *I*_Kr_ and *I*_Ks_ activation and produces a seemingly paradoxical shortening of APD_90_ in SR,^26^ a finding replicated in our study in aEHT. However, even under this condition, *I*_Kr_ dominates over *I*_Ks_ in our *in silico* model so substantially that *I*_Ks_ has no considerable contribution to repolarization. Predictions were confirmed by experimental data in SR but also in aEHT, qualifying both models as helpful to study human atrial electrophysiology.

#### Contribution of *I*_Ks_ to repolarization in atrial fibrillation

Situation is different in AF, where APD_20_ is much longer and plateau voltage clearly above 0 mV, a value substantially above the *V*_0.5_ activation of *I*_Ks_ reported here. Nevertheless, activation of *I*_Ks_ in AF is very small. Computer models are an established tool in cellular cardiac electrophysiology.^41^ Obviously, computer models should recapitulate experimental observations. Reasons for the mentioned above discrepancy remain open but illustrate that the present *in silico* models for AF-induced remodelling may need further refinement. It is hard to decide if the model has failed or if the data used to feed the model were imperfect. We have not measured tachypacing-remodelled aEHT since at present, beating rate in atrial EHTs is high (∼3 Hz in this study), close to the rate used for tachypacing.^6^

#### Relevance of *I*_Ks_ for human atrium vs. ventricle: is β-adrenoceptor-responsiveness of *I*_Ks_ the crucial point?

The transcript levels for α subunits mediating *I*_Ks_ and *I*_Kr_ are similar in atrial and ventricular tissues.^23^ From this finding, one could expect relevance of *I*_Ks_ and *I*_Kr_ in both chambers. However, LQT2 (mutation in *I*_Kr_) but not LQT1 (mutation in *I*_Ks_) has an impact on AF in humans. We suspect that lack of β-AR stimulation to increase *I*_Ks_ could play a role. The lack of isoprenaline to increase *I*_Ks_ in atrial CM is perplexing and could be interpreted that our cells (both aCM and hiPSC-aCM) lack β-AR receptor and/or other key proteins of cAMP/PKA signalling. Such an assumption seems very unlikely in the case of adult human atrial CM, where β-AR stimulation increases cAMP, *I*_Ca_, and force.^42–44^ The situation is less clear for hiPSC-aCM. However, from our own unpublished work, it is evident that hiPSC-aCMs show a consistent increase in *I*_Ca_ upon β-AR stimulation. We suspect that lack of effect of isoprenaline on *I*_Ks_ could be a common finding in atrial tissue, since we could also not detect an increase in *I*_Ks_ upon isoprenaline in guinea pig atrial cardiomyocytes (data not shown). No data are available on β-AR responsiveness of *I*_Ks_ in aCM from other species. In isolated dog ventricular CM, stimulation of β-AR increases *I*_Ks_ and β-AR stimulation alone is sufficient to unmask *I*_Ks_ contribution to repolarization.^24^ The situation in human ventricle is more complex since two interventions (β-AR stimulation and *I*_Kr_ block), simultaneously applied, were needed to unmask *I*_Ks_ contribution to repolarization.^35,45^ Therefore, it was concluded that β-AR stimulation is mandatory to unmask *I*_Ks_ contribution in human ventricles.

Despite our long-time experience, we still fail to record stable AP in atrial tissue exposed to β-AR agonists when potassium channels (*I*_Kr_ or *I*_Kur_) are blocked. Thus, it remains open if stimulation of β-AR in the presence of *I*_Kr_ block may allow detection of *I*_Ks_ contribution to repolarization in aEHT and native human atrial tissue.

#### Atrial engineered heart tissue as a model to study human atrial electrophysiology

In contrast to ventricular EHTs,^45^ we cannot slow down beating rate in aEHT, complicating interpretation of APD data. Importantly, APD_90_ in fast beating aEHT (basal condition) was not shorter than in atrial tissue (SR) when paced at 3 Hz.^27^ Nevertheless, aEHTs^8^ share electrophysiological properties of human atrium (shortening of APD upon muscarinic receptor activation and prolongation of APD_20_ but shortening of APD_90_ upon blocking of *I*_Kur_). We feel confident that aEHTs recapitulate key findings in contribution of delayed rectifier potassium current to repolarization in the human atrium. *I*_Ks_ block was without an effect on APD_90_, and fast beating should have facilitated contribution of *I*_Ks_ to repolarization. On the other hand, blocking of *I*_Kr_ prolonged APD_90_ to an extent not different in effect size compared with human SR tissue.

#### Translational perspective

Our findings do not support the idea that *I*_Ks_ should contribute to APD shortening in AF.^32^ The lack of β-AR stimulation–mediated increase in *I*_Ks_ in the human atrium might explain why LQT1 associates with ventricular but not with atrial arrhythmias. Importantly, hiPSC-aCM recapitulates key findings on *I*_Ks_ in the human atrium and may therefore qualify them as an interesting model to study human atrial electrophysiology.

## Limitations

Chronic treatment of the patients with beta-blockers may have interfered with the results obtained both from isolated cells and in atrial trabeculae. The small number of patients not treated with β-blockers (3 out of 37) precludes a meaningful interpretation. However, we would not expect that chronic treatment of the patients with beta-blockers may have masked response of *I*_Ks_ to isoprenaline since β-blocker treatment increases β-AR sensitivity in atrial tissues from patients.^46^ This partly reverses G_i_ alpha-upregulation and β-AR downregulation in heart failure.^47^

AP measurements in human atrial tissue may not be accurate enough to detect very small increases in APD_90_ by 1.5% as predicted in some of our model simulations. For technical reasons, we have measured AP in right atrial tissues at low pacing rate (1 Hz). aEHT beats much faster (3.1 ± 0.2 Hz). In ventricular EHT (generated without RA hiPSC-CM), even low concentration (300 nM) of ivabradine slowed beating below 1 Hz.^45^ In aEHT, even very high concentration of ivabradine slowed down beating rate only by about 25% (own unpublished data). Thus, at present, we cannot slow beating rate in aEHT substantially. Due to shortage in AF tissue, we cannot provide experiments with HMR-1556 in the presence of E-4031.

## Supplementary Material

euae140_Supplementary_Data

## Data Availability

All relevant data are within the manuscript and its online supplementary material.
