# Supplementary material for: Human induced pluripotent stem cell–derived atrial cardiomyocytes recapitulate contribution of the slowly activating delayed rectifier currents IKs to repolarization in the human atrium
Source: Europace. 2024 May 24;26(6):euae140. doi: 10.1093/europace/euae140 (PMC11167676; doi:10.1093/europace/euae140)
Supplement: euae140_Supplementary_Data [file euae140_supplementary_data.zip › I_Ks_Supplement Figures_R1 .pptx]

## Slide 1
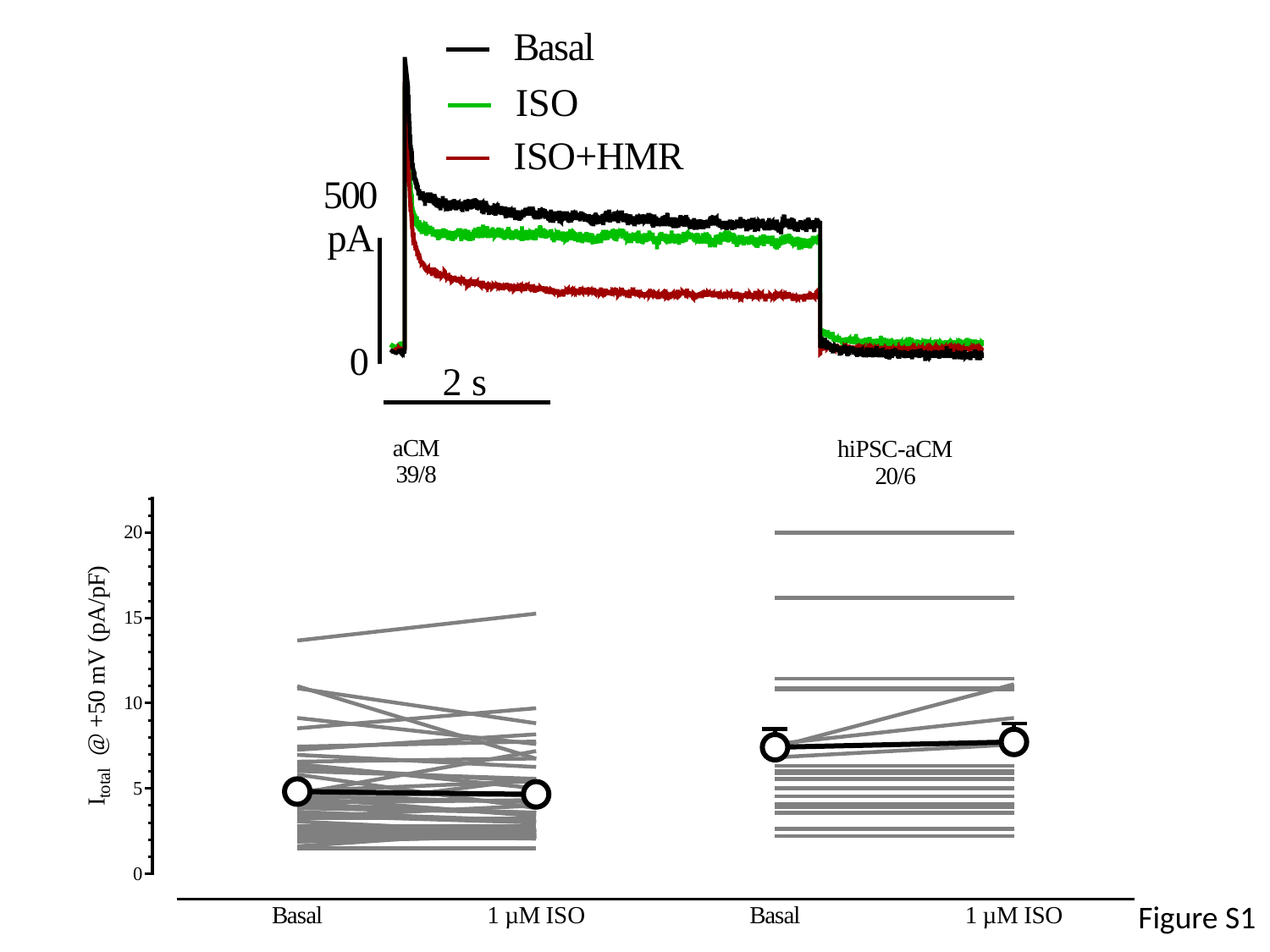

Figure S1

## Slide 2
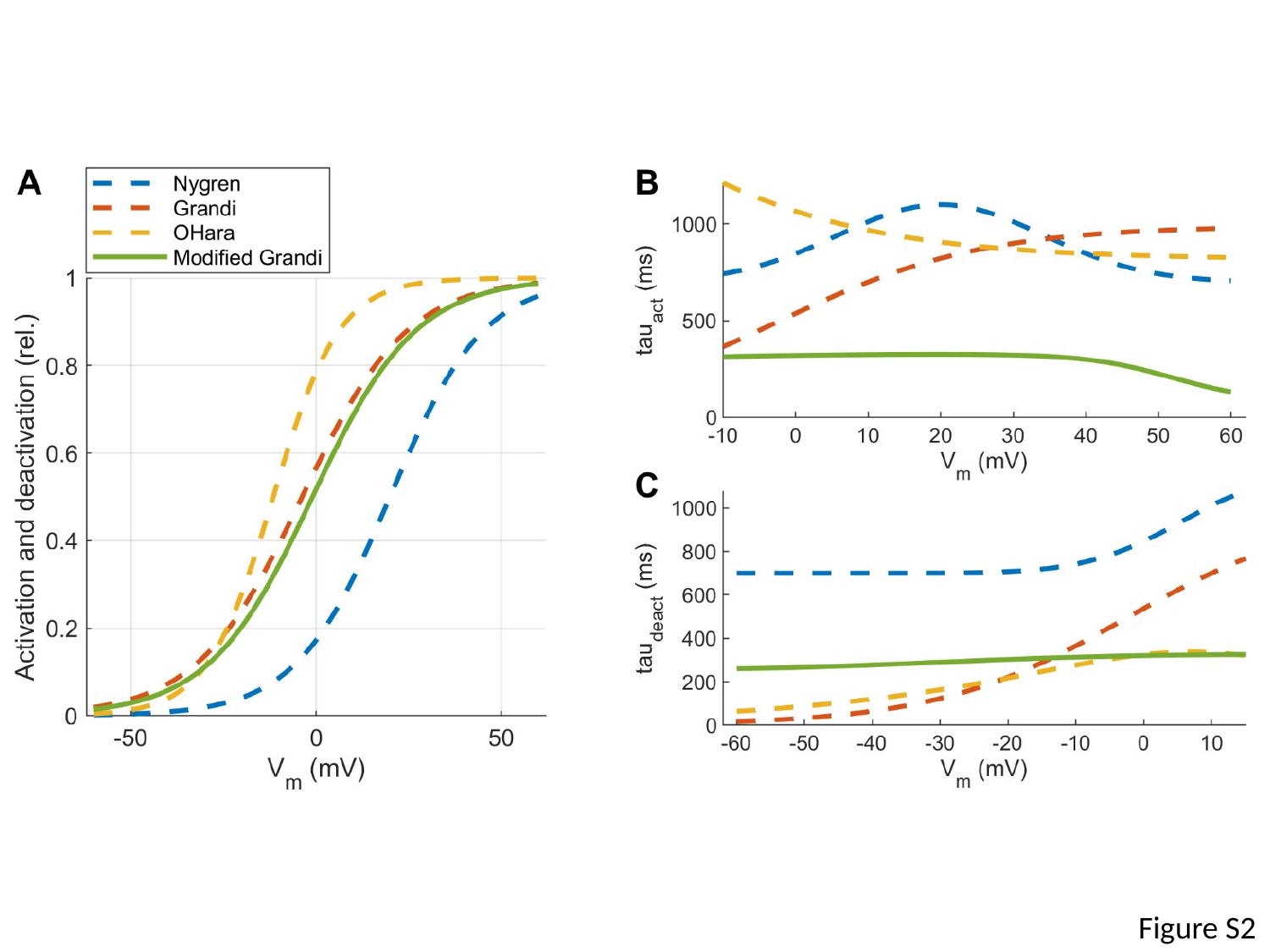

Figure S2

## Slide 3
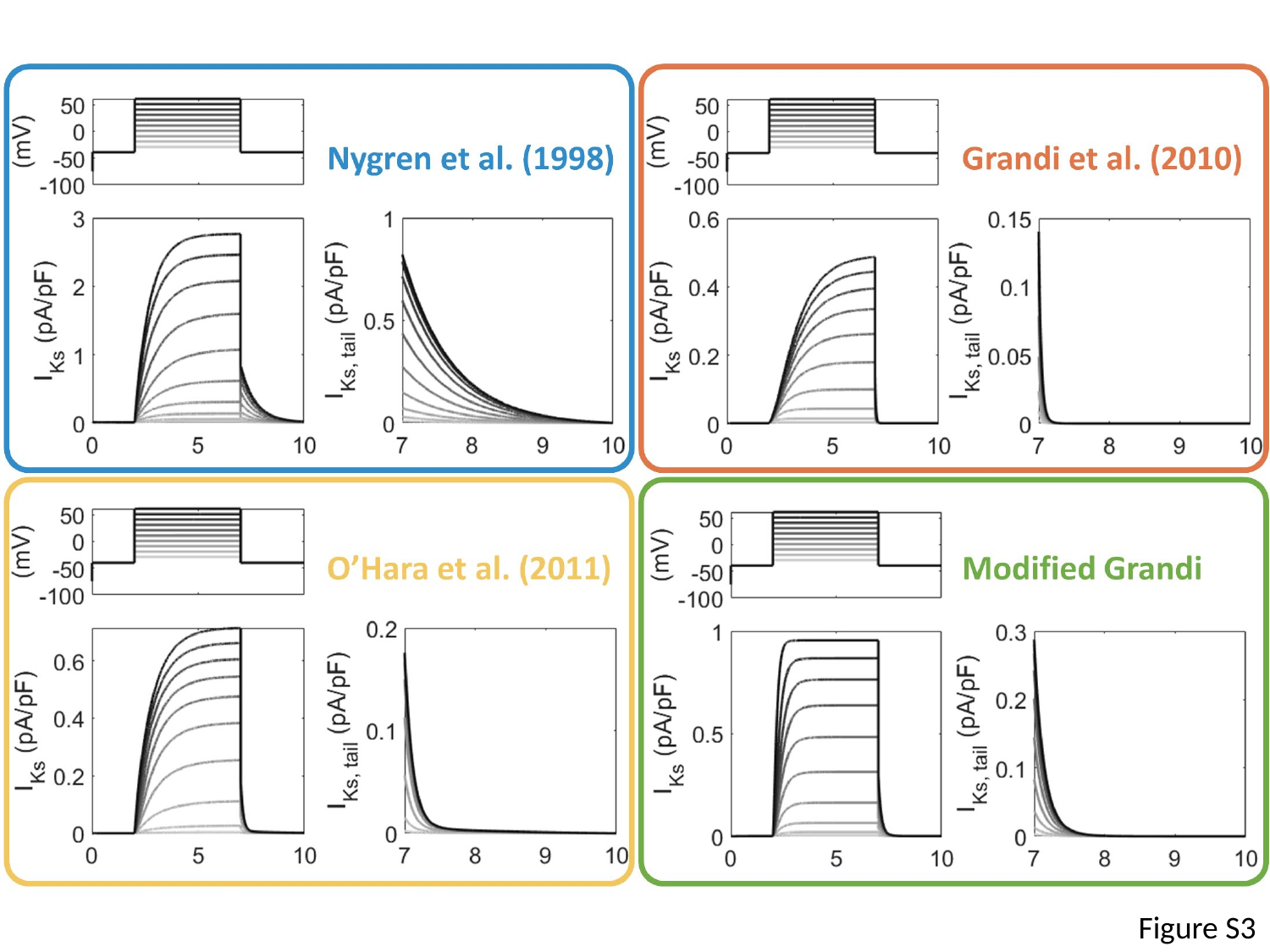

Figure S3

## Slide 4
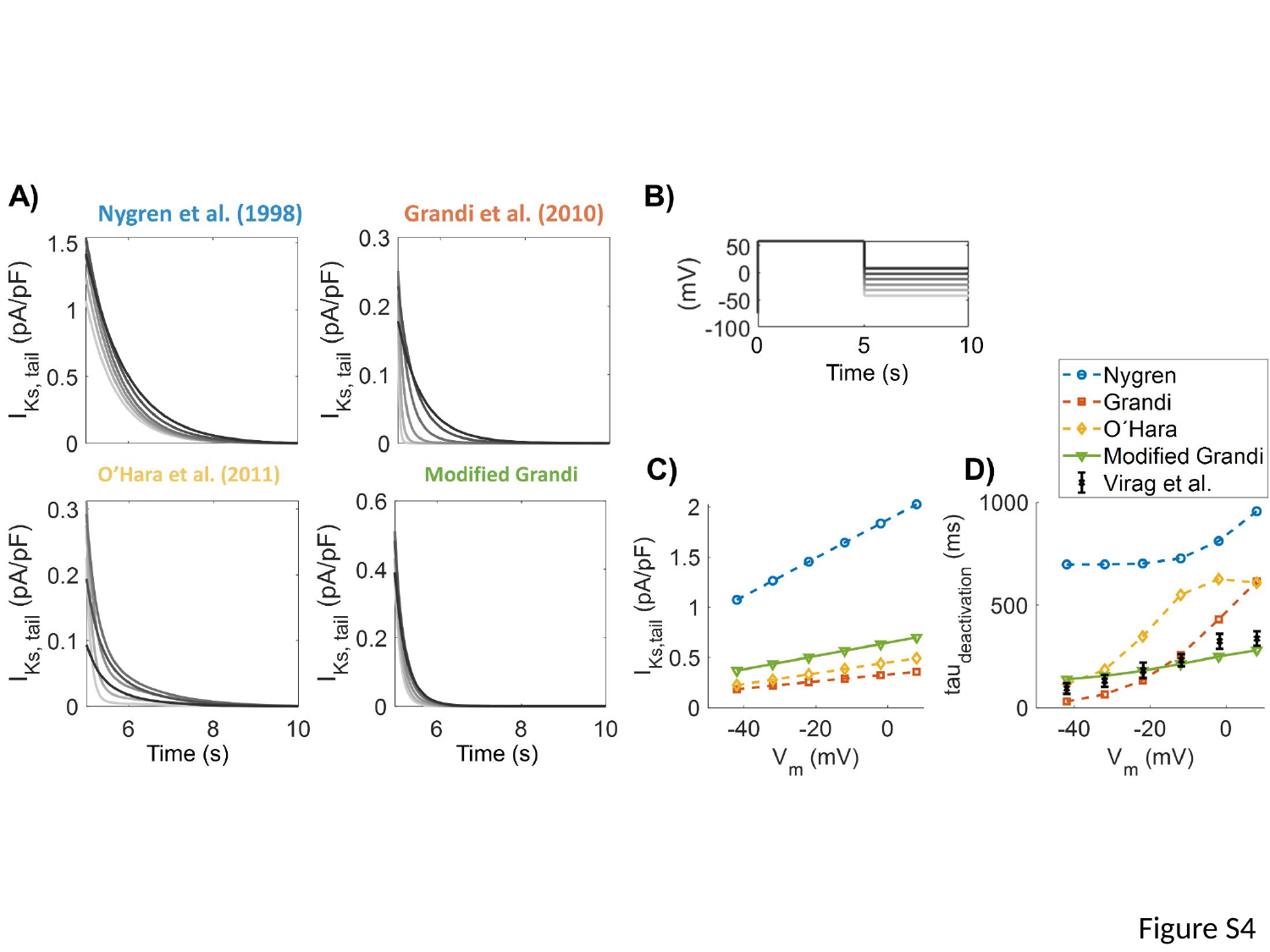

Figure S4

## Slide 5
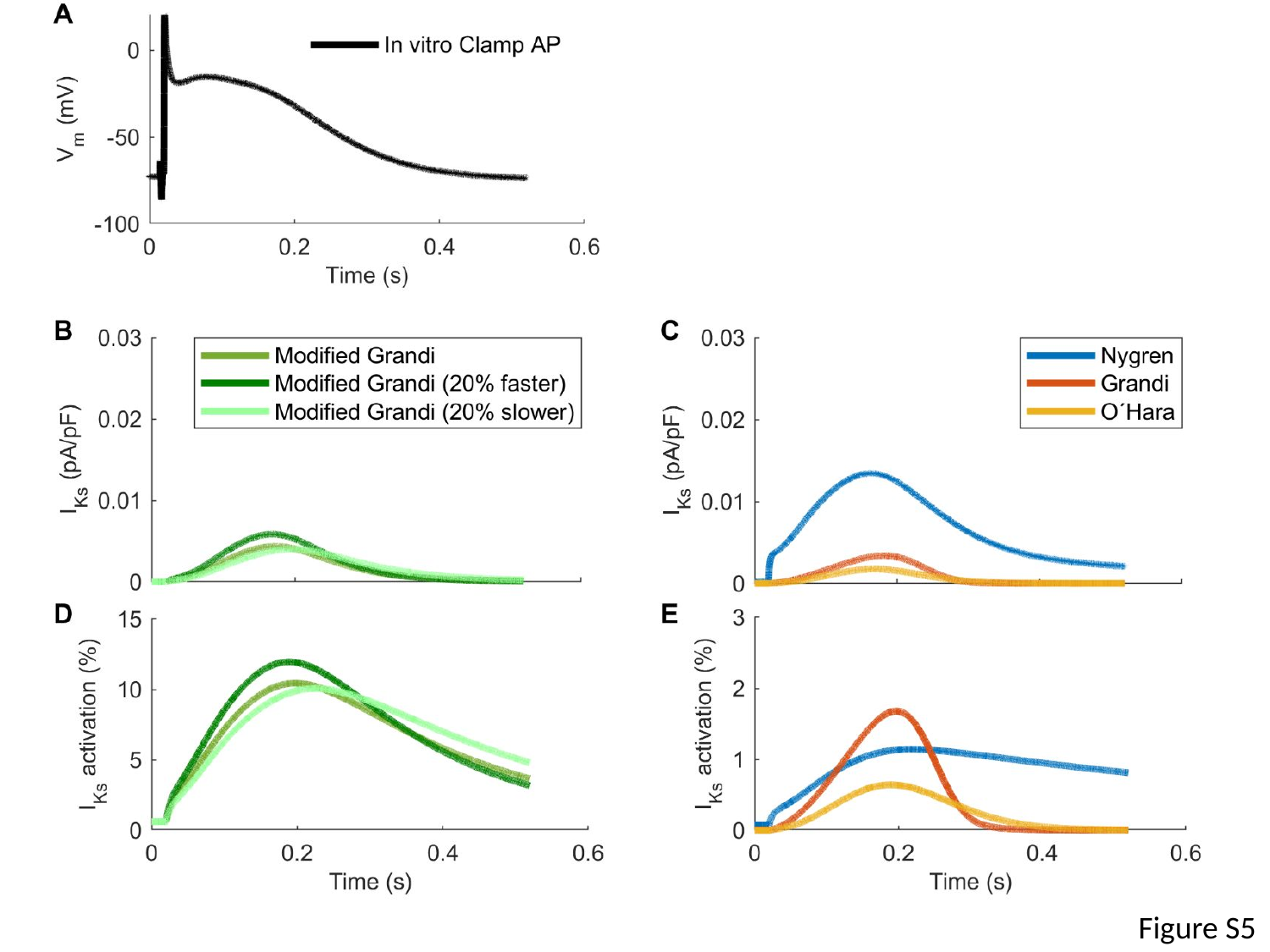

Figure S5

## Slide 6
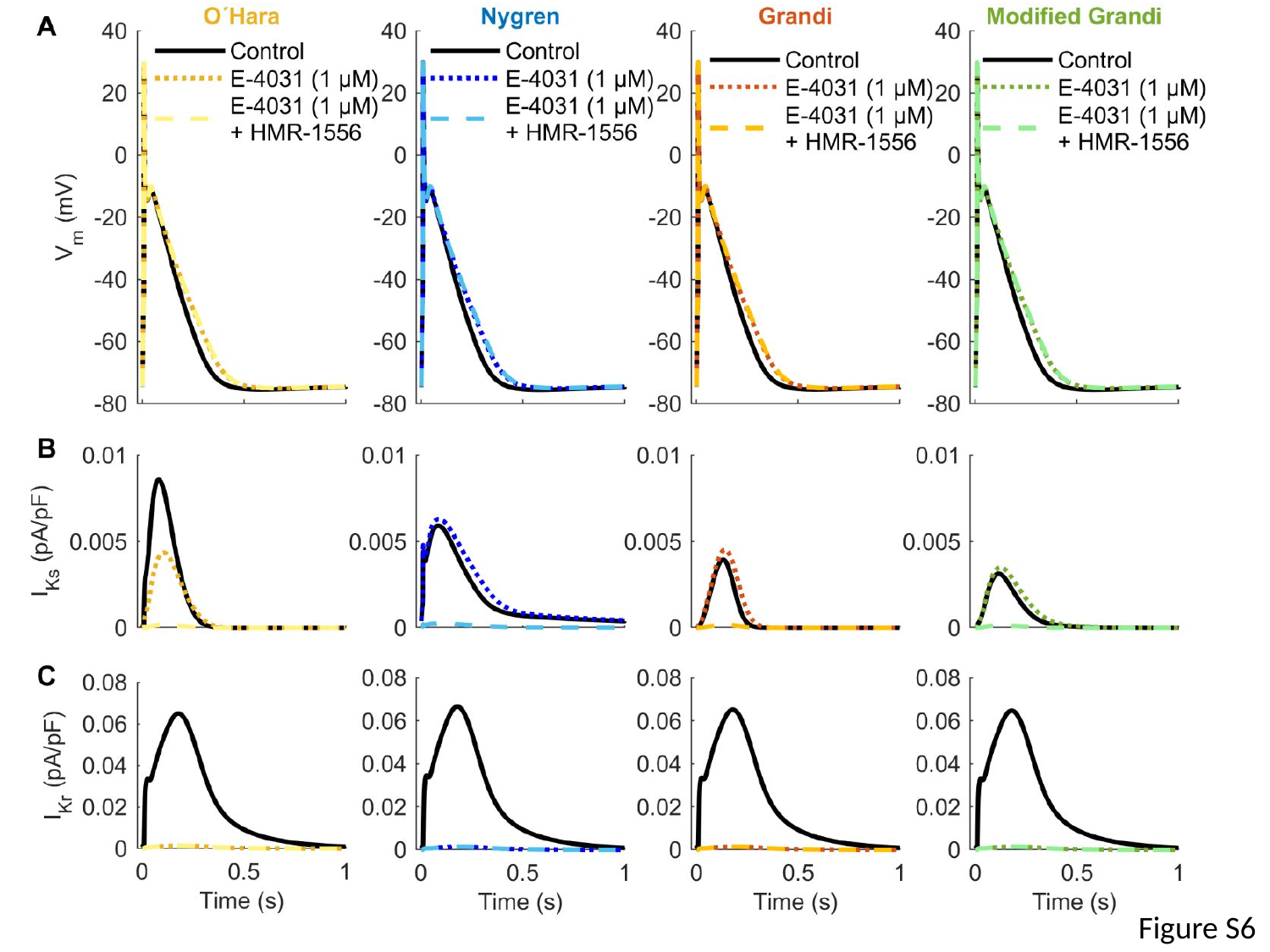

Figure S6

## Slide 7
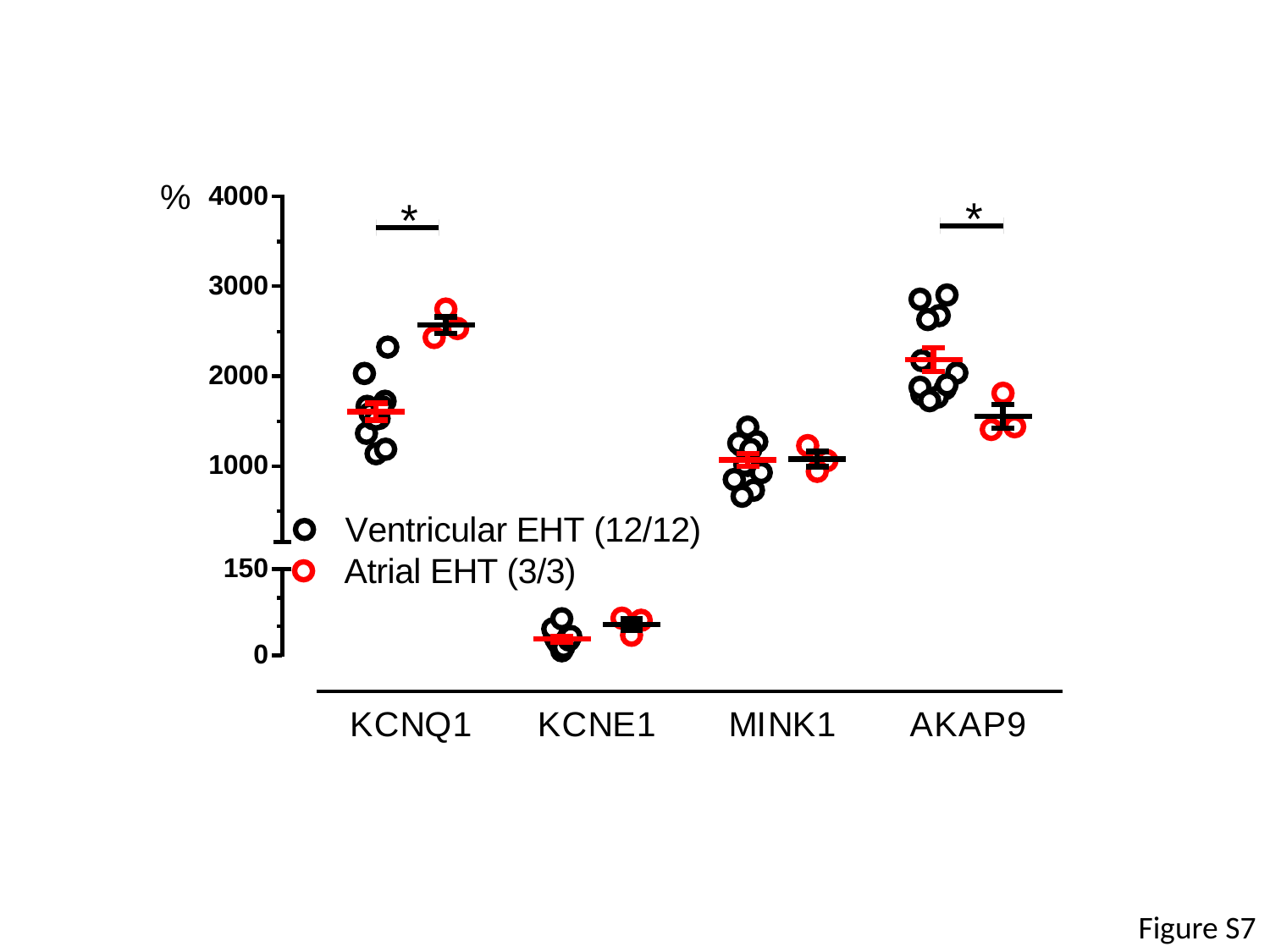

Figure S7
