# Supplementary material for: Human induced pluripotent stem cell–derived atrial cardiomyocytes recapitulate contribution of the slowly activating delayed rectifier currents IKs to repolarization in the human atrium
Source: Europace. 2024 May 24;26(6):euae140. doi: 10.1093/europace/euae140 (PMC11167676; doi:10.1093/europace/euae140)
Supplement: euae140_Supplementary_Data [file euae140_supplementary_data.zip › I_Ks_Supplement methods and legends_R1 .docx]

**Supplementary methods***1. Updated in silico cell model and simulation protocols*

In the present study, we made modifications to our previously published human atrial cardiomyocyte (aCM) model (1) based on our experimental data and studies found in literature. Modifications were made to slow, rapid and ultra-rapid delayed potassium currents (I_Ks_, I_Kr_ and I_Kur_) and chronic/persistent atrial fibrillation (cAF) model variant. In addition, new drug options recapitulating the *in vitro* experiments were introduced to the model. All details are given below.

*1.1. Description of I_Ks_ kinetics in previous in silico models*

Previously published models have taken different approaches to formulations of I_Ks_ kinetics. The aCM models by Nygren et al. (2) and Courtemanche et al.(3) have based their I_Ks_ formulations on experimental data of aCM recorded by Wang *et al*. (4). The aCM model by Grandi et al (5) used the same formulation for I_Ks_ as in their previously published ventricular cell model (6). Their model parameterisation is based on experimental data obtained from human ventricular myocytes by Virag et al. (7). The same data has been used for formulating I_Ks_ in ventricular myocyte model by O’Hara et al. (8). However, they opted to use separate equations for time constants of activation and deactivation, opposed to Nygren et al. model (2) and Grandi et al (5) whose formulations employ one equation to describe both processes.
Here, we took the I_Ks_ formulation by Grandi et al. model (5) as a starting point. We modified the parameters that define the voltage dependence and developed a new formulation for the time constant for activation and deactivation of I_Ks_. The properties of the new I_Ks_ formulation and its function as part of the whole aCM model were compared the previous formulations in multiple conditions (Figures 3 and S2-S5).

*1.2. Reparameterised I_Ks_*

We developed a new formulation for I_Ks_ to better match the new *in vitro* data, regarding voltage- and time-dependent properties of the current. The new formulation was compared to the one by Grandi et al. (5) below (please, see also Figure S2):

% Grandi et al.

gKs = 0.175 ;

Xs_ss = 1 / (1+exp(-(V_m + 3.8)/14.25));

Xs_tau = 0.9901 / (1+exp(-(V_m + 2.436)/14.12));

IKs = gKs * Xs^2 * (V_m - E_K);

% new formulation

gKs = 0.34;

Xs_ss = 1 / (1 + exp(-(V_m + 1.0)/14));

IKsntau = 0.72 / (1 + exp((V_m - 48)/10) + exp(-(V_m - 48)/150));

IKs = gKs * Xs^2 * (V_m - E_K);

*1.3. Modifications to I_K_ and I_Kur_ in the aCM model*

We adjusted the conductances of I_Kr_ and I_Kur_ to improve the spike and dome action potential (AP) shape and to better match the current block effects on AP morphology observed *in vitro*. Specifically, the conductances of I_Kr_ and I_Kur_ were multiplied by 1.3 and 0.8, respectively.

*1.4. The approach to account for AF-related remodeling and the minor update*

To model the effect chronic/persistent atrial fibrillation (cAF) *in silico*, we used our previously published holistic scheme (1,9) that accounts for the remodeling sarcolemmal ion currents, intracellular calcium handling and cellular hypertrophy. The only update to the cAF model variant that we did in this study was the adjustment of I_Ks_ multiplication parameter value. It was changed from 2.45 to 1.97 to match the cAF-related change in I_Ks_ amplitude that was shown in our new *in vitro* data.

*1.5. Simulation of drug impacts on ion currents*

The new drug options in the *in silico* model employ the same simple pore block model that is commonly used (10)(. Briefly, the effect of a drug on an ion channel was implemented by scaling the max conductance according to the drug concentration and half-maximal inhibitory concentration:

g([D]) = g_max_ / (1 + [D]/IC_50_).

The new drug conditions are as follows:

- **4-AP:** the IC_50_ values for I_to_ and I_Kur_ have been reported to be 1.0 mM and 5.8 μM, respectively (11).
- **HMR-1556:** the affected ion currents are I_CaL_, I_to_, I_Kr_ and I_Ks_. IC_50_ used in the model are 27.5 μM, 33.9 μM, 12.5 µM, and 20 nM, respectively (12)
- **E-4031 + HMR-1556:** the drug modifications of E-4031 and HMR-1556 in the model are combined. Since both affect I_to_, the IC_50_ having the stronger inhibitory effect was used.
- **4-AP + HMR-1556:** The drug modifications of 4-AP and HMR-1556 in the model are combined. Since both affect I_Kr_, the smaller IC_50_ value was used.

*1.6. Simulation protocols used in the study*

At the beginning of every simulation, the starting values were obtained by running a simulation without a stimulus for a duration of 60 minutes. This quiescent steady-state reached at the end of the simulation was used as the starting state for the pacing simulation. Unless stated otherwise, external ion concentrations of 130 mM, 5.4 mM and 1.8 mM were used in the simulations for sodium (Na_ext_), potassium (K_ext_) and calcium (Ca_ext_), respectively.

We accounted for the liquid junction potentials that affect the *in vitro* patch clamp measurements of ion currents by shifting the voltages used in this study and in the Virag et al. (7) study by +11 and +8 millivolts, respectively.

In Figure 3, the *in silico* data was obtained with voltage clamp protocol. The *in silico* model was first clamped to holding potential of -40 mV for 2 seconds, after which the clamping voltage was changed to test voltage for 5 seconds, and finally back to holding potential of -40 mV for 3 seconds. Ten test potentials were used, starting from -29 mV and increasing by increments of 10 mV. The data obtained during applying the test voltage was used for creating the figure. In the simulation, a Ca_ext_ concentration of 2.0 mM was used.

In Figure 4A, pacing protocol was used, in which the *in silico* model was first paced for 5 minutes with BCL of 1000 ms to reach quasi steady-state. The stimulus current had an amplitude of -49 nA/nF, and duration of 5 ms. The presented *in silico* data was then obtained by pacing the model with the same BCL and stimulus for the duration of one AP cycle of 1000 ms.

In Figure 4A, the simulation protocol was identical to Figure 4B.

In Figure S5, the simulation protocol was identical to Figure 4A, except for the stimulus amplitude that was -108.6624 nA/nF.

In Figure S2, time constant of I_Ks_ in different models were plotted as a function of membrane voltage for comparison.

In Figure S3, the voltage clamp protocol was identical to Figure 3.

In Figure S4, similar voltage clamp protocol was used as described in (7). The *in silico* model was first clamped to holding potential of 50 mV for 5 seconds, after which the clamping voltage was changed to test voltage for 5 seconds. Six test potentials were used, starting from -42 mV and increasing by increments of 10 mV. BCL of 2000 ms was used. The external ion concentrations used for Na_ext_, K_ext_ and Ca_ext_ were 144.33 mM, 4.0 mM, and 1.8 mM, respectively.

In Figure S6, *in silico* data was obtained by using an AP trace measured *in vitro* as a voltage clamp for the hA-CM model, employing the four different I_Ks_ formulations.

***References***

1. Skibsbye L, Jespersen T, Christ T, Maleckar MM, van den Brink J, Tavi P, et al. Refractoriness in human atria: Time and voltage dependence of sodium channel availability. J Mol Cell Cardiol [Internet]. 2016;101:26–34. Available from: http://dx.doi.org/10.1016/j.yjmcc.2016.10.009

2. Nygren A, Fiset C, Firek L, Clark JW, Lindblad DS, Clark RB, et al. Mathematical model of an adult human atrial cell: The role of K+ currents in repolarization. Circ Res. 1998;82(1):63–81.

3. Courtemanche M, Ramirez RJ, Nattel S. Ionic mechanisms underlying human atrial action potential properties: Insights from a mathematical model. Am J Physiol - Hear Circ Physiol. 1998;275(1 44-1).

4. Wang Z, Fermini B, Nattel S. Rapid and slow components of delayed rectifier current in human atrial myocytes. Cardiovasc Res. 1994;28(10):1540–6.

5. Grandi E, Pandit S V., Voigt N, Workman AJ, Dobrev D, Jalife J, et al. Human atrial action potential and Ca 2+ model: Sinus rhythm and chronic atrial fibrillation. Circ Res. 2011;109(9):1055–66.

6. Grandi E, Pasqualini FS, Bers DM. A novel computational model of the human ventricular action potential and Ca transient. J Mol Cell Cardiol [Internet]. 2010;48(1):112–21. Available from: http://dx.doi.org/10.1016/j.yjmcc.2009.09.019

7. Virág L, Iost N, Opincariu M, Szolnoky J, Szécsi J, Bogáts G, et al. The slow component of the delayed rectifier potassium current in undiseased human ventricular myocytes. Cardiovasc Res. 2001;49(4):790–7.

8. O’Hara T, Virág L, Varró A, Rudy Y. Simulation of the undiseased human cardiac ventricular action potential: Model formulation and experimental validation. PLoS Comput Biol. 2011;7(5).

9. Koivumäki JT, Seemann G, Maleckar MM, Tavi P. In Silico Screening of the Key Cellular Remodeling Targets in Chronic Atrial Fibrillation. PLoS Comput Biol. 2014;10(5).

10. Brennan T, Fink M, Rodriguez B. Multiscale modelling of drug-induced effects on cardiac electrophysiological activity. Eur J Pharm Sci. 2009;36(1):62–77.

11. Amos GJ, Wettwer E, Metzger F, Li Q, Himmel HM, Ravens U. Differences between outward currents of human atrial and subepicardial ventricular myocytes. J Physiol. 1996;491(1):31–50.

12. Gogelein H, Bruggemann A, Gerlach U, Brendel J, Busch AE. Inhibition of I(Ks) channels by HMR 1556. Naunyn Schmiedebergs Arch Pharmacol [Internet]. 2000 [cited 2023 Jan 9];362(6):480–8. Available from: https://link.springer.com/article/10.1007/s002100000284

**Figure S1: Stimulation of β-adrenoceptors did not increase I_Ks_ in adult SR-aCM and hiPSC-aCM**

**Top:** Original traces of outward currents at the end of the 5s-long test pulse to +50 mV before and after adding 1 µM isoprenaline in hiPSC-aCM. **Bottom:** Outward current densities measured at +50 mV in adult SR-aCM and hiPSC-aCM before (Basal) and after adding 1µM isoprenaline (ISO). Gray lines indicate individual cells, circles mean±SEM. Data are given only for cells with I_Ks_, as confirmed by exposure to HMR-1556. *p<0.05, paired t-test vs. basal, numbers indicate number of cells/patients or cells/EHT.

**Figure S2: Comparison of the mathematical formulations for slow delayed rectifier potassium current.**

Labeling: Nygren = Nygren et al. (1998), Grandi = Grandi et al. (2010), O’Hara = O’Hara et al. (2011), and modified Grandi = rescaled and shifted version of the Grandi et al. (2010) formulation. **A**: Voltage dependence of the steady-state activation (Nygren, Grandi, and modified Grandi) and activation&deactivation (O’Hara). **B** and **C**: Voltage dependence of the activation and deactivation time constants.

**Figure S3: Raw simulation data corresponding to the in silico results shown in Figure 3.**

**Figure S4: Validation of the new current formulation modified from Grandi et al.**

Simulation results obtained with the different mathematical formulations of the slow delayed rectifier potassium current are compared to the characteristics measured in vitro by Virag et al. (2001). **A**: raw simulation data from the in silico voltage clamp experiments. **B:** Voltage clamp protocol. **C:** Amplitude of the tail current. **D:** Time constant of deactivation for the tail current.

**Figure S5: Comparison of I_Ks_ characteristics using the in vitro action potential as voltage clamp in simulations.**

**A:** Action potential trace measured in vitro used as a voltage clamp in simulations. **B:** Changing the time constant of the modified Nygren I_Ks_ formulation (±20%) had a relatively small impact on the simulated current trace. **C:** I_Ks_ current trace simulated with the other formulations. **D and E:** Relative activation or availability of the channel for the model versions in (**B**) and (**C**).

**Figure S6:** **Comparison variant of the computational cell models simulated drug effects in sinus rhythm**

**A:** Impact of E-4031, E-4031 + HMR-1556 on the action potential morphology in O`Hara, Nygren, Grandi and modified Grandi models. **B-C**: Underlying changes in the slow and rapid activating delayed rectifier potassium currents

**Figure S7:** **Expression Ks channel proteins and regulatory subunits in atrial/ventricular EHT**

Summary of gene expressions in ventricular (n=12/12) and atrial EHT (n=3/3). Units (%) for all expression values are 2^-ΔCt^ versus reference gene (×100) expressed. Mean±SEM, n/n indicates number of EHT/number of batches. * indicates P-value <0.05 ventricular vs. atrial EHT groups (paired test following ANOVA).

**Supplement Table 1: Action potential characteristics in SR, in AF and aEHT**

Summary of AP characteristics under basal conditions in SR and AF (paced at 1 Hz) and in aEHT beating spontaneously at 3.1±0.2 Hz, Mean±SEM, n/n indicates number of tissues/number of patients or number of EHT/number of batches in case of atrial EHTs, * indicates P-value <0.05 vs. basal (paired test following ANOVA).

**Supplement Table 2. Effects of I_Kur_, I_Kr_ and I_Ks_ block on action potential duration (APD_90_) in human RAA (SR and AF), aEHT and in silico models (SR and AF).**

Summary of changes in APD_90_ (expressed as % of control in case 4-AP and E-4031 or in % of 4-AP or E-4031 when HMR-1556 was added on top. Mean±SEM, n/n indicates number of tissues/number of patients or number of EHT/number of batches in case of atrial EHTs, unpaired test following ANOVA.

**Supplement Table 3. Effects of I_Kur,_ I_Kr_ and I_Ks_ block on action potential in silico models**

Summary of AP characteristics *in silico* model under basal condition, I_Ks_ block (HMR-1556), I_Kur_ block (4-AP) and I_Kr_ block (E-4031) presence or absence I_Ks_ block. Parameters are diastolic membrane potential (V_diast_), sistolic membrane potential (V_syst_), amplitude of action potential (V_ampl_), maximum upstroke velocity (V_max_), action potential duration at that 90%, 50% repolarization (APD_50_ and APD_90_).
