# Supplementary material for: Human induced pluripotent stem cell–derived atrial cardiomyocytes recapitulate contribution of the slowly activating delayed rectifier currents IKs to repolarization in the human atrium
Source: Europace. 2024 May 24;26(6):euae140. doi: 10.1093/europace/euae140 (PMC11167676; doi:10.1093/europace/euae140)
Supplement: euae140_Supplementary_Data [file euae140_supplementary_data.zip › I_Ks_Supplement Tables_R1 .docx]

**Supplement Table 1: Values of basal action potential in SR, in AF and aEHT (3.1±0.2 Hz)**

|  | **SR (n=10/10)** | **aEHT (n=11/4)** | **AF (n=7/7)** |
| --- | --- | --- | --- |
| RMP/MDP (mV) | -71.5±1.1 | -72.4±2.2 | -76.5±1.1^#^ |
| APA (mV) | 93.5±2.0 | 93.3±4.2 | 98.8±1.5 |
| V_max_ (V/s) | 184.9±20.8 | 154.8±17.1 | 170.0±15.4 |
| APD_20_ (ms) | 6.7±2.3 | 8.7±1.1 | 28.7±6.5* |
| APD_50_ (ms) | 114.0±16.4 | 43.9±3.5* | 91.7±4.9 |
| APD_90_ (ms) | 289.7±13.4 | 169.1±12.7* | 201.4±8.5* |
| V_Plateau_ (mV) | -17.3±1.3 | -27.0±2.3* | -6.2±4.6* |

Summary of AP characteristics under basal conditions. Parameters are resting membrane potential (RMP) for SR and AF, maximum diastolic potential (MDP) for EHT, amplitude of action potential (APA), maximum upstroke velocity (V_max_), action potential duration at that 90%, 50%, 20% repolarization (APD_20_, APD_50_, APD_90_), plateau (V_Plateau_). Mean±SEM, n/n indicates number of tissues/number of patients or number of EHT/number of batches in case of atrial EHTs, * indicates P-value <0.05 SR vs. aEHT or AF (paired test following ANOVA). ^#^ indicates P-value <0.05 SR vs. AF (unpaired t-test).

**Supplement Table 2. Effects of I_Kur,_ I_Kr_ and I_Ks_ block on action potential duration (APD_90_) in human right atrial appendices of patients in sinus rhythm (SR) or atrial fibrillation (AF), aEHT and in silico models (SR and AF).**

| **Change in APD90 (in %)** | **SR** | **SR in Silico** | **Atrial EHT** | **AF** | **AF in Silico** |
| --- | --- | --- | --- | --- | --- |
| 4-AP | -9.37±3.6  (6/6) | -2.5 | -23.2±5.6*  (5/2) | 11.4±5.5  (7/7) | 21.3 |
| HMR-1556 on top of 4-AP | -3.0±1.9  (6/6) | 4.3 | 3.9±6.3  (5/2) | 3.9±0.8  (7/7) | 17.1 |
| E-4031 | 19.8±2.7  (4/4) | 16.7 | 44.6±11.2*  (6/2) | n.d. | 30.5 |
| HMR-1556 on top of E-4031 | -2.0±2.9  (4/4) | 1.5 | 1.4±3.9  (6/2) | n.d. | 1.8 |

Summary of changes in APD_90_ expressed as % of control in case 4-AP and E-4031 or in % of 4-AP or E-4031 when HMR-1556 was added on top. Mean±SEM, n/n indicates number of tissues/number of patients or number of EHT/number of batches in case of atrial EHTs, *: SR vs atrial EHT, unpaired test following ANOVA.

**Supplement Table 3. Effects of I_Kur,_ I_Kr_ and I_Ks_ block on action potential *in silico* models**

|  | Basal | HMR-1556 | 4-AP | 4-AP+HMR-1556 | E-4031 | E-4031+HMR-1556 |
| --- | --- | --- | --- | --- | --- | --- |
| \| V_diast_ (mV) \| -75.6 \| -75.5 \| -77.5 \| -77.1 \| -75.1 \| -75.0 \| \| --- \| --- \| --- \| --- \| --- \| --- \| --- \| | -75.6 | -75.5 | -77.5 | -77.1 | -75.1 | -75.0 |
| V_syst_ (mV) | 29.1 | 29.1 | 30.2 | 30.0 | 30.0 | 29.7 |
| V_ampl_ (mV) | 104.7 | 104.6 | 107.7 | 107.1 | 105.1 | 104.7 |
| V_max_ (V/s) | 170.5 | 171.4 | 172.3 | 172.7 | 175.9 | 173.8 |
| APD_50_ (ms) | 91.0 | 91.9 | 199.2 | 208.8 | 93.8 | 94.8 |
| APD_90_ (ms) | 295.6 | 301.1 | 288.1 | 308.4 | 345.0 | 349.3 |

Summary of AP characteristics *in silico* model under basal condition, I_Ks_ block (HMR-1556), I_Kur_ block (4-AP) and I_Kr_ block (E-4031) presence or absence I_Ks_ block. Parameters are diastolic membrane potential (V_diast_), systolic membrane potential (V_syst_), amplitude of action potential (V_ampl_), maximum upstroke velocity (V_max_), action potential duration at that 90%, 50% repolarization (APD_50_ and APD_90_).
